# A Review of DEtection TRansformer: From Basic Architecture to Advanced Developments and Visual Perception Applications

**DOI:** 10.3390/s25133952

**Published:** 2025-06-25

**Authors:** Liang Yu, Lin Tang, Lisha Mu

**Affiliations:** College of Software Engineering, Sichuan Polytechnic University, Deyang 618000, China; scpubullhorse@scpu.edu.cn (L.Y.); tlwylwolf@scpu.edu.cn (L.T.)

**Keywords:** object detection, DETR, transformer, attention, end to end, deep learning

## Abstract

DEtection TRansformer (DETR) introduced an end-to-end object detection paradigm using Transformers, eliminating hand-crafted components like anchor boxes and Non-Maximum Suppression (NMS) via set prediction and bipartite matching. Despite its potential, the original DETR suffered from slow convergence, poor small object detection, and low efficiency, prompting extensive research. This paper systematically reviews DETR’s technical evolution from a “problem-driven” perspective, focusing on advancements in attention mechanisms, query design, training strategies, and architectural efficiency. We also outline DETR’s applications in autonomous driving, medical imaging, and remote sensing, and its expansion to fine-grained classification and video understanding. Finally, we summarize current challenges and future directions. This “problem-driven” analysis offers researchers a comprehensive and insightful overview, aiming to fill gaps in the existing literature on DETR’s evolution and logic.

## 1. Introduction

Object detection, a fundamental task in computer vision (CV), aims to localize and identify object instances within images. It is also a significant research direction within the field of Artificial Intelligence (AI). Deep learning has greatly propelled the development of this field [1,2]. From two-stage methods represented by R-CNN [2] and Faster R-CNN [3] to one-stage methods like the YOLO series [4,5,6,7,8] and SSD [9], detection accuracy and speed have continuously improved. However, these methods, which have long held a dominant position, generally rely on a series of hand-designed components and heuristic strategies [10]. Specifically, the commonly used anchor box mechanism, which uses predefined prior boxes with preset object sizes and aspect ratios, is not only highly sensitive to hyperparameters but also struggles to adapt to complex and diverse object shapes and flexible cross-domain scenarios. Furthermore, it often leads to severe issues of positive-negative sample imbalance and computational redundancy [11]. In addition, two-stage methods depend on complex Region Proposal Networks (RPNs) to generate candidate regions, increasing procedural complexity. Most mainstream object detection methods use NMS [12] as a post-processing method to remove redundant bounding boxes. NMS is essentially a greedy algorithm that is threshold-sensitive and prone to suppressing correct results when dealing with dense or occluded objects [13]. More critically, as a separate step, it breaks the end-to-end trainability of the original detection pipeline, thereby hindering the joint optimization of the model. These inherent limitations brought about by core components collectively constitute a bottleneck for improve traditional object detection methods, making the exploration of a new, simpler, more effective, and truly end-to-end paradigm an urgent need in the field.

The Transformer architecture, initially demonstrating remarkable success in Natural Language Processing (NLP) [14], emerged as a compelling alternative to Convolutional Neural Networks (CNNs) due to the inherent strengths of its self-attention mechanism in modeling long-range dependencies and enabling parallel computation. This prompted a fundamental inquiry within the Computer Vision (CV) community: Could Transformer’s powerful sequence modeling capabilities be adapted for visual tasks? Unlike the intrinsic inductive biases of CNNs, such as locality and translation equivariance, Transformer offers a more generalized approach to global context modeling, which is crucial for understanding complex inter-element relationships in images [15]. Although early attempts integrated attention mechanisms into CNNs [16,17,18], they generally served as auxiliary components rather than replacing the core convolutional structure.

**Figure 1:**
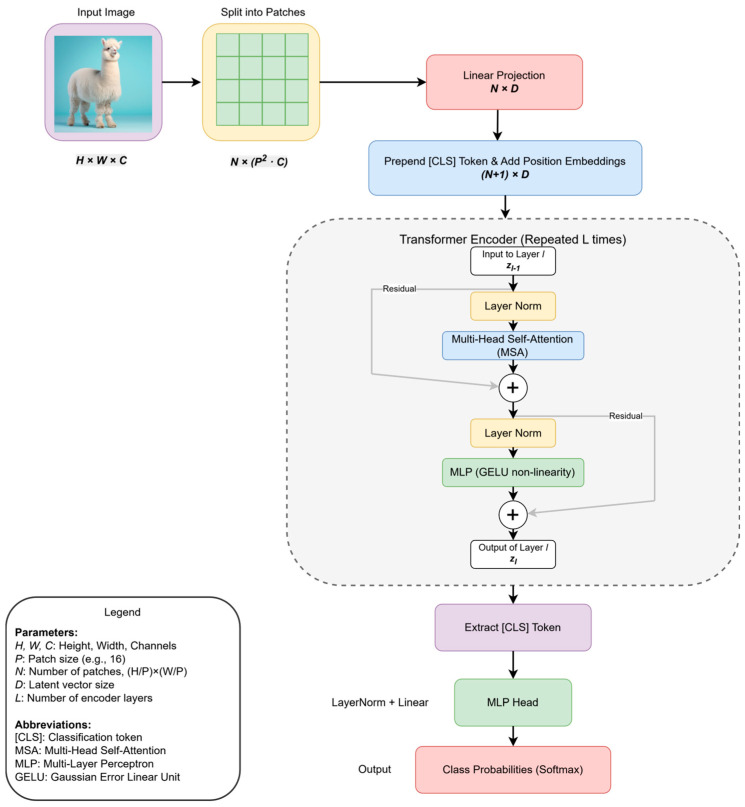
Vision Transformer (ViT) network architecture.

The pivotal Vision Transformer (ViT) provided a definitive affirmative answer, establishing a new paradigm for image processing. Its network architecture, detailed in Figure 1, re-frames image recognition as a sequence-to-sequence problem. As depicted, ViT begins by partitioning an input image into a sequence of non-overlapping patches. These patches are then linearly projected into a D-dimensional embedding space, creating a sequence of patch embeddings. To retain spatial information, which is otherwise lost in the sequence representation, learnable position embeddings are added to the patch embeddings. Furthermore, a special [CLS] (classification) token is prepended to the sequence, for which its final output state is used to represent the entire image for the classification task.

**Figure 2:**
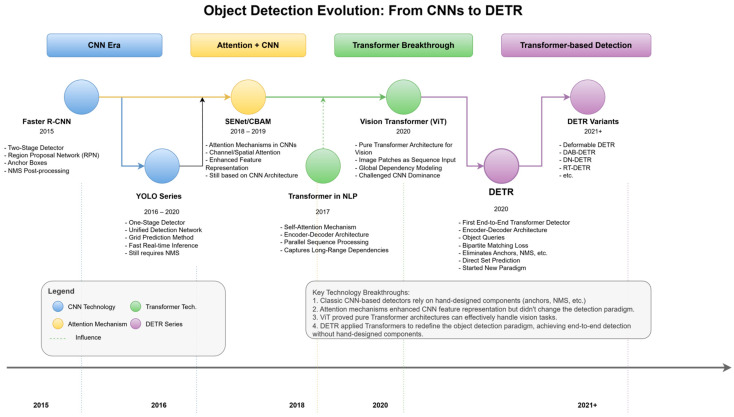
Object detection evolution: from CNNs to DETR.

This complete sequence is then fed into a standard Transformer encoder, which is repeated L times. Each encoder block consists of a multi-head self-attention (MSA) module and a multi-layer perceptron (MLP), with layer normalization (LN) and residual connections applied around each module. Finally, for the classification task, the state of the [CLS] token at the output of the Transformer encoder is passed through an MLP head, which yields the final class probabilities, typically via a softmax function. By demonstrating that a pure Transformer architecture could achieve state-of-the-art results on large-scale image recognition benchmarks, ViT fundamentally challenged the long-standing dominance of CNNs in computer vision. This success ignited a surge of research interest in applying Transformer-based models to more complex CV tasks [19,20,21], thereby laying the foundational groundwork for their subsequent integration into the field of object detection. To more clearly illustrate the technical evolution, Figure 2 depicts the key stages in the object detection field’s gradual transition from traditional CNN-based architectures to Transformer-based architectures.

Against this backdrop, Carion et al. proposed DETR [22], a landmark study in object detection. This work was the first to fully apply the standard Transformer encoder–decoder architecture to object detection, completely discarding anchor boxes, RPN, and NMS. It cleverly redefined the object detection task as a set prediction problem, directly predicting an unordered set containing all objects (categories, bounding boxes, etc.) for a given image. The core of this paradigm shift lies in its innovative set loss mechanism based on bipartite matching, which uses the Hungarian algorithm [23], to find a unique ground truth box for each predicted box for supervision, thus achieving true end-to-end training. DETR achieved performance comparable to optimized Faster R-CNN baselines on the COCO dataset [24] experiments, particularly excelling in large object detection. However, DETR’s success came with corresponding costs: Firstly, its convergence was relatively slow, often requiring over 500 training epochs, far exceeding traditional CNN-based methods that typically only need between 50 and 300 epochs [25]; secondly, its performance on small object detection tasks was suboptimal [26]; finally, its computational efficiency was lower, with inference speeds (especially on CPU) significantly slower than Faster R-CNN [27].

Since its proposal in 2020, DETR has rapidly developed within just a few years, giving rise to large numbers of subsequent studies and variant models. Currently, while some of the literature has summarized specific aspects of DETR, there is a lack of a comprehensive review that systematically analyzes how its core challenges have catalyzed key technical breakthroughs from a “problem-driven” perspective, clarifies its adaptation and expansion in different application domains, and fully envisions future directions. After nearly five years of development, the DETR technical ecosystem has taken initial shape, with key challenges and mainstream solutions gradually becoming clear. At this juncture, a systematic review and outlook are crucial for researchers in the field to accurately grasp the technical evolutionary path and gain insight into future trends. This review is based on this background, aiming to fill this gap and provide readers with an in-depth understanding of the DETR technical landscape.

We aim to systematically review the development trajectory of DETR since its inception. Our core contribution lies in providing a “problem-driven” analysis that delves into how the core challenges of the original DETR have catalyzed key technical breakthroughs and the evolution of detection models, detailing their manifestations in areas such as attention mechanisms, query design, training strategies, and model architecture. We will extensively discuss the core innovations and trade-offs embodied in milestone models like Deformable DETR [11], DN-DETR [28], and DINO [29]. We analyze the applications and challenges of DETR in critical domains such as autonomous driving and medical imaging, summarize the current technical bottlenecks, and envision future research directions. Through this “problem-driven” analysis, we hope to provide researchers, engineers, and graduate students with a systematic understanding of the evolution of the DETR architecture, key technical bottlenecks and solutions, and its application potential in different visual perception tasks, with a deep understanding of the evolutionary path of the DETR technical ecosystem, revealing its continuous potential as a general visual perception framework.

**Figure 3:**
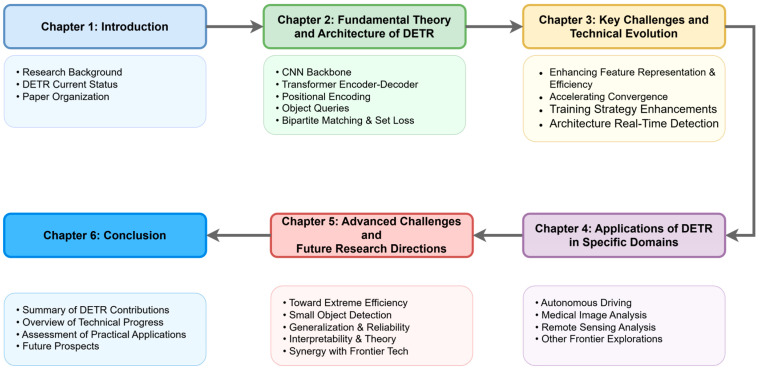
A review of DETR (detection transformer): from basic architecture to advanced developments and visual perception applications.

To achieve the aforementioned goals, we will adopt a structured framework of “Problem Formulation—Solutions—Application Expansion—Advanced Challenges & Future Outlook”, as shown in Figure 3. We believe that this organizational approach, centered around the development and evolutionary path of DETR itself, can most clearly reveal the inherent logic of its technical evolution and future potential, thereby providing unique value to readers. Through this organizational approach, we first conduct a “problem-driven” review of the key technical evolutions (Section 3). We then demonstrate the practical impact of these advancements by examining their application in key domains (Section 4). Building on this foundation of both technical progress and practical application, we finally shift to a forward-looking analysis of the advanced challenges and future research directions that lie ahead (Section 5). This clear progression from key technical advancements to applications in various domains and then to future research is central to this paper’s narrative.

The structure of this paper is arranged as follows.

Section 2 provides a detailed exposition of DETR’s fundamental theory and core architecture, including the CNN backbone, Transformer encoder–decoder, positional encoding, object queries, and the crucial bipartite matching and set prediction loss mechanism. Following this, Section 3, from a “problem-driven” perspective, delves into the key technical evolutions spurred by DETR’s initial core challenges (slow convergence, poor small object performance, and low efficiency), systematically reviewing milestone improvements in areas like attention mechanisms, query designs and supervision methods, and architectural optimizations. Section 4 then discusses the applications of DETR and its variants in key specific domains such as autonomous driving, medical image analysis, and remote sensing image analysis, analyzing how to adapt to domain challenges and demonstrating application potential. Building on this, Section 5 delves into the advanced challenges still facing the DETR ecosystem and outlines the key future research directions that stem from them. Finally, Section 6 summarizes the entire paper, reiterating DETR’s significant contributions, key technical advancements, and its expansive future potential as a foundational perception framework.

## 2. Fundamental Theory and Architecture of DETR

**Figure 4:**
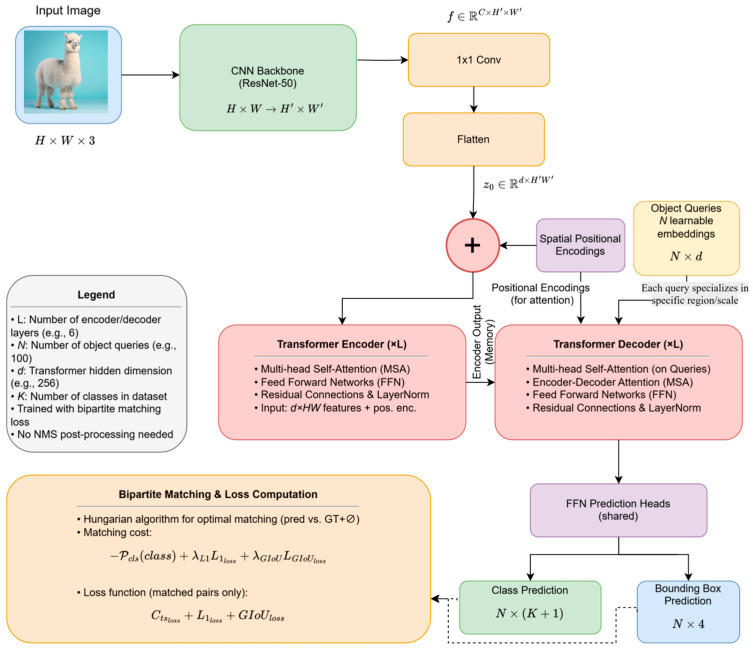
DETR network architecture.

DETR’s architectural design cleverly integrates the standard Transformer encoder–decoder structure with the requirements of object detection, constructing a concise and powerful end-to-end framework. This section will detail its core components and key mechanisms. The basic network architecture of DETR is shown in Figure 4.

### 2.1. Backbone: Extracting Image Features

**Figure 5:**
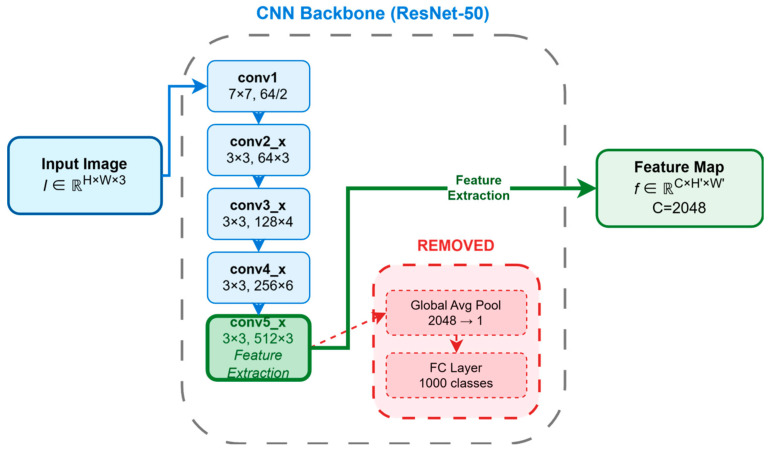
CNN backbone feature extraction process.

The processing pipeline of DETR begins with a standard CNN backbone. Similarly to many detectors, it utilizes a CNN (e.g., ResNet-50 or ResNet-101 [30] pretrained on ImageNet [31]) to extract rich and hierarchical structured visual features from the input image $I\in {\mathbb{R}}^{H\times W\times 3}$. The processing pipeline of DETR begins with a standard CNN backbone. Similarly to many modern detectors, it utilizes a CNN (e.g., ResNet-50 or ResNet-101 [31]) that has been pretrained on a large-scale image classification dataset, most notably ImageNet [30]. The ImageNet datasets, a vast repository containing over 14 million manually annotated images across more than 20,000 categories, have become a standard benchmark for developing and pre-training deep learning models in computer vision. By training on such a massive and diverse dataset, the CNN backbone learns to recognize a rich and generalizable hierarchy of visual patterns—from fundamental features like edges and textures to more complex object parts and concepts. This pre-training process endows the model with a powerful feature representation that serves as an excellent initialization for downstream tasks. When this pre-trained backbone is applied to object detection, these learned features can be effectively transferred, which significantly accelerates model convergence and boosts the final detection performance. The global average pooling layer and classification layer at the end of the CNN are removed, and the output from a deeper stage $f\in {\mathbb{R}}^{C\times H^{\prime }\times W^{\prime }}$ is used as the starting point for subsequent processing. The modification of the standard CNN backbone is illustrated in Figure 5. The choice of CNN as the feature extractor is based on two considerations: Firstly, it effectively leverages the powerful pretrained weights and mature structures of CNNs [32,33]; secondly, CNNs are proficient at capturing local patterns and spatial hierarchical information [34,35,36]. However, to obtain more powerful semantic representations, the spatial resolution of the DETR output feature map $H^{\prime }\times W^{\prime }$ is typically quite low (downsampled by a factor of 32 relative to the input H×W), but its channel dimension C is large (up to 2048). While this low-resolution characteristic is advantageous for capturing global information, it is not conducive to fine-grained spatial information capture. This is one of the key reasons why the original DETR algorithm performs poorly in detecting small objects [37]. Before this feature map is fed into the Transformer, its high channel dimension is reduced, and its spatial structure is flattened. Specifically, a 1 × 1 convolutional layer is first applied to the feature map f to reduce its channel dimension from C down to the Transformer’s required hidden dimension d, resulting in an intermediate feature map of size $d\times (H^{\prime }W^{\prime })$. Subsequently, this map is flattened along its spatial dimensions to create the final input sequence for the encoder, denoted as $z_{0}\in {\mathbb{R}}^{d\times (H^{\prime }W^{\prime })}$. This sequence is then ready for input into the Transformer encoder.

### 2.2. Positional Encoding: Injecting Spatial Information

**Figure 6:**
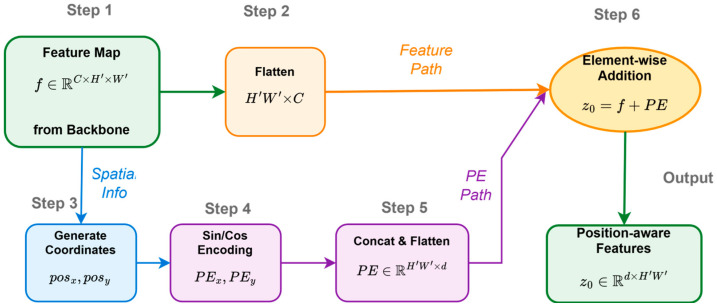
Positional encoding mechanism in DETR.

To enable the permutation-invariant Transformer model to understand the spatial origin of each element in the input feature sequence, the introduction of positional encoding is crucial. Similarly to the original Transformer model, DETR also employs fixed sine/cosine encoding. It independently generates encoding vectors for the x and y coordinates of the feature map and combines them. For a coordinate value pos (typically representing x or y) and an encoding dimension index i, the encoding formula is as follows:

(1) $$\begin{array}{l} PE(pos,2i)=\sin(pos/10000^{\frac{2i}{d}});\\ PE(pos,2i+1)=\cos(pos/10000^{\frac{2i}{d}}). \end{array}$$

where PE represents the positional encoding vector, and d is the model’s embedding dimension. By using the sine and cosine functions of different frequencies to generate encodings for different dimensions, the model can infer relative positional information through the linear transformations of the encoding vectors and generalize the feature maps of different sizes. These positional encodings are added element-wise to the flattened feature sequence $z_{0}$, forming the input to the encoder. Figure 6 provides a visual representation of this process, showing how spatial coordinates are transformed into positional encodings and subsequently fused with the image features. More importantly, positional encoding also explicitly participates in the computation of all attention layers in the encoder and decoder, typically by adding it to the query and key embedding vectors, allowing the attention mechanism to perceive the spatial origin of features and perform position-aware feature aggregation. Although subsequent research has explored alternatives such as conditional spatial queries [38], fixed sine/cosine encoding is the basic design of DETR. While this fixed, frequency-based positional encoding method is simple and effective, it has certain limitations. For example, this design only encodes absolute positional information and has limited ability to express relative positional relationships. Furthermore, its extrapolation ability may be limited when processing feature maps that are significantly larger than the training data. The fixed encoding method may also be insufficient for adapting to different scales, rotations, or more complex geometric transformations. Additionally, sharing the same encoding for all positions may not fully capture more detailed spatial priors specific to certain tasks or datasets. These limitations have inspired the exploration of more flexible or learnable positional encoding schemes in subsequent research.

### 2.3. Transformer Encoder: Enhancing Global Context

**Figure 7:**
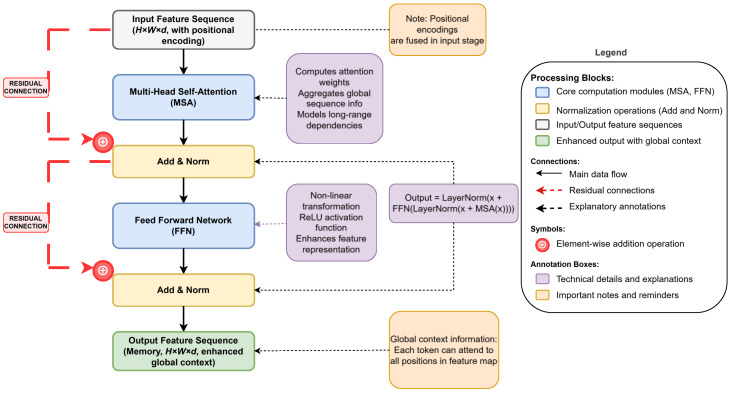
Structure of a single Transformer encoder layer.

After obtaining the high-dimensional feature sequence fused with positional information, the Transformer encoder undertakes the core task of further processing these features and capturing global contextual dependencies. The encoder is composed of L (L = 6 in the original Transformer) standard Transformer encoder layers stacked together. The structure of each encoder layer is standard. To provide a more intuitive illustration of the internal structure and data flow within the Transformer encoder layer, Figure 7 presents a comprehensive architecture of a single encoder layer. This diagram clearly demonstrates the specific roles and interconnections of multi-head self-attention mechanisms, feed-forward networks, and residual connections in the feature processing pipeline. Through this visualization, we can better understand how the encoder progressively transforms input image feature sequences into enhanced feature representations with global contextual information. The residual connections and layer normalization operations shown in the figure ensure the stable training of deep networks, while the multi-head self-attention mechanism enables each position to attend to all other positions in the feature map, effectively modeling long-range dependencies. In the following sections, we will provide a detailed analysis of each component of the encoder layer in conjunction with Figure 7. First, through a multi-head self-attention (MSA) mechanism module, each token in the input sequence (corresponding to a spatial location in the original feature map) is allowed to attend to all other tokens in the sequence, effectively modeling long-range dependencies within the image by computing and aggregating attention-weighted features; then, a feed-forward network (FFN) is used for non-linear feature transformations. Each sub-layer utilizes residual connections and layer normalization (LN) [39] to aid training. After processing the encoder layers by L, the output feature sequence $memory\in {\mathbb{R}}^{d\times (H^{\prime }W^{\prime })}$ contains feature representations enhanced with global contextual information, which serve as the basis for the decoder to query objects.

### 2.4. Transformer Decoder: Querying and Decoding Objects

The context-rich image features output by the encoder provide the foundation for object decoding. The Transformer decoder utilizes this set of features while introducing a set of learnable object queries to jointly complete the final object localization and identification task. DETR is also composed of L layers, but its design revolves around querying these N objects. These queries are D-dimensional embedding vectors independent of the input image, and they are randomly initialized at the beginning of training. These queries can be considered as N “slots” that gradually learn to act as proxies for specific object instances during training. The characteristic of these object queries being randomly initialized independent of the input image content makes it difficult for the model to establish a stable association between queries and specific image regions or objects in the early stages of training. This is a significant reason for DETR’s slow convergence. Understanding the learning dynamics of object queries and optimizing their design is one of the core directions of subsequent DETR variant research. A notable feature of DETR’s decoder is that it processes these N queries in parallel. Each decoder layer contains three key sub-layers (it also uses residual connections and LN to aid training).

**Figure 8:**
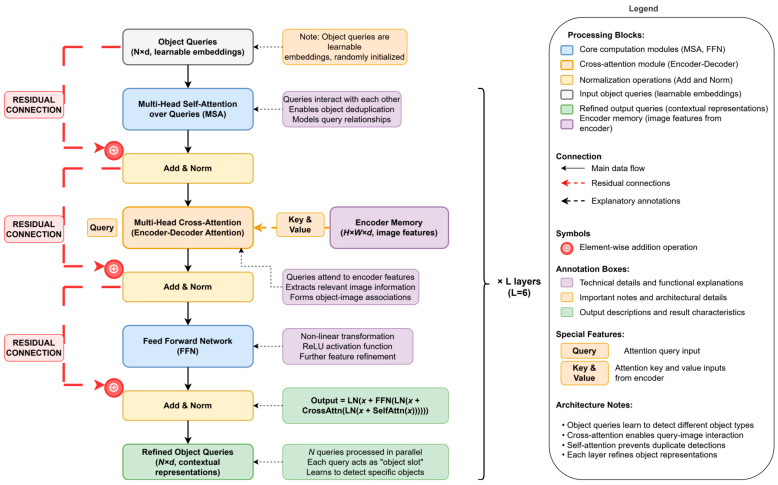
Internal structure of Transformer decoder layer.

To provide a clearer illustration of the internal architecture and data flow within the Transformer decoder layer, Figure 8 presents a comprehensive structure of a single decoder layer. This diagram clearly demonstrates how object queries are progressively refined through three key sub-layers: The multi-head self-attention mechanism enables queries to interact with each other, the cross-attention mechanism facilitates effective fusion between queries and encoder output features, and the feed-forward network further enhances feature representations. The residual connections and layer normalization operations shown in the figure ensure the stable training of deep networks, while the N learnable object queries are processed in parallel, with each query acting as an “object slot” that gradually learns to detect specific object instances. Through this visualization, we can better understand how the decoder transforms randomly initialized object queries into contextual object representations rich in semantic information.

1. Self-Attention over Queries: MSA is applied to the N object queries, allowing the queries to interact with each other and perceive the objects they might be attending to, thereby helping the model avoid generating duplicate detection boxes and serving as an implicit deduplication mechanism.
2. Cross-Attention: This is crucial for decoding object information. It uses the object queries from the previous layer as the query and the image features output by the encoder as the key and value. Through the cross-attention mechanism (also called encoder–decoder attention), each object query can effectively query the encoded image feature map, attend to image regions related to its potential object, and aggregate the corresponding feature information to update its own representation.
3. FFN: Its structure and function are similar to the FFN in the encoder, performing further feature transformation on the output of the cross-attention. The object queries are passed layer by layer through the L decoder layers and are progressively refined. The final output $q_{out}\in {\mathbb{R}}^{N\times d}$ is the representation learned by the decoder for these N potential objects, containing key information used for the subsequent prediction of their categories and locations.

### 2.5. Prediction Heads: Generating Final Predictions

**Figure 9:**
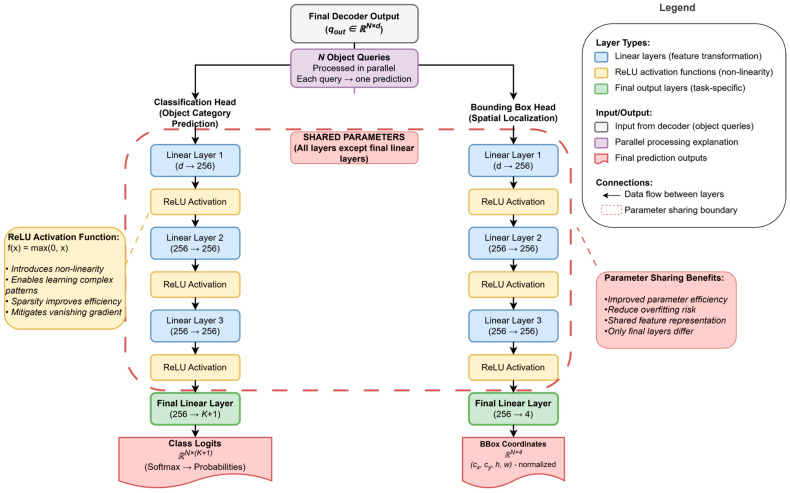
DETR prediction head architecture.

The N query embeddings $q_{out}$ obtained after multi-layer refinement by the decoder have encoded potential object information. To transform this information into concrete detection results, they are fed into two prediction heads with shared parameters. These two heads are typically simple MLP structures and share all parameters except the last layer to improve parameter efficiency. Their output is directly used for subsequent set prediction loss calculations. The original DETR uses an MLP containing three hidden layers with the rectified linear unit (ReLU) activation function, followed by one linear layer. The ReLU activation function, defined as f(x)=max(0,x), is a critical non-linear component in modern neural networks. Its primary role is to introduce non-linearity into the model, enabling it to learn more complex patterns from the data than a simple linear model could. By setting all negative values to zero, ReLU helps make the network sparse, which can be computationally efficient. Furthermore, it effectively mitigates the vanishing gradient problem, which often plagues other activation functions like Sigmoid, thereby allowing for the faster and more stable training of deep networks. The functions of these two prediction heads are as follows. To provide a clearer illustration of the internal architecture and data processing flow of DETR prediction heads, Figure 9 presents the complete structure of both prediction heads. This diagram clearly demonstrates how the N query embeddings from the decoder’s output are processed through two parallel multi-layer perceptrons (MLPs): the classification head for predicting object categories and the bounding box head for spatial localization. The figure particularly emphasizes the parameter-sharing mechanism—all layers except the final linear layers are shared between the two heads, which effectively improves parameter efficiency and reduces overfitting risk. The introduction of ReLU activation functions provides the necessary non-linearity for the network to learn complex feature representations. Through this visualization, we can better understand how DETR transforms abstract query embeddings into concrete detection results.

1. Classification Head: Responsible for predicting the category corresponding to each query. It outputs K+1 dimensional logits. After passing through a Softmax function, it yields the probability distribution of each query belonging to one of the K object categories or the “no object” background class.
2. Bounding Box Head: Responsible for predicting the bounding box corresponding to each query. It outputs four real values, directly regressing the normalized bounding box center coordinate $\left(c_{x},c_{y}\right)$ and the box height (h) and width (w). These coordinates are relative to the entire image’s size.

### 2.6. Set Prediction Loss and Bipartite Matching: The Key to End-to-End Training

To achieve end-to-end training without anchor boxes, RPN, and NMS, DETR introduces its most iconic innovation: the set prediction loss based on bipartite matching. Its core idea is to treat object detection as a set prediction problem, where the model needs to predict a set of N elements {yi^} and match and supervise it with a set of M ground truth objects $\left\{y_{i}\}(M\leq N\right)$ (padded with N−M background symbols ∅ relative to size N). Specifically, DETR uses the classic Hungarian algorithm to find the optimal one-to-one matching σ between the predicted set and the ground truth set. This optimal matching aims to minimize the total cost of all N matched pairs, considering both the similarity of category predictions and bounding box predictions. For a prediction yi^=(pi^,bi^) and a ground truth object $y_{j}=(c_{i},b_{i})$ (where p^ is the class probability, b is the bounding box, and c is the ground truth class), the cost function is defined as

(2) $$C_{match}({\hat{y}}_{i},y_{j})=\left\{\begin{array}{c} -\lambda_{cls}\cdot {\hat{p}}_{\sigma (i)}(c_{j})+\lambda_{L1}\cdot {\left\|{\hat{b}}_{i}-\left.b_{\sigma (i)}\right\|\right.}_{1}+\lambda_{GIoU}\cdot L_{GIoU}(b_{\sigma (i)},{\hat{b}}_{j}),\;\mathrm{if}\;j\neq \emptyset ;\\ \;0\;\;\;\;\mathrm{if}\;j=\emptyset . \end{array}\right.$$

**Figure 10:**
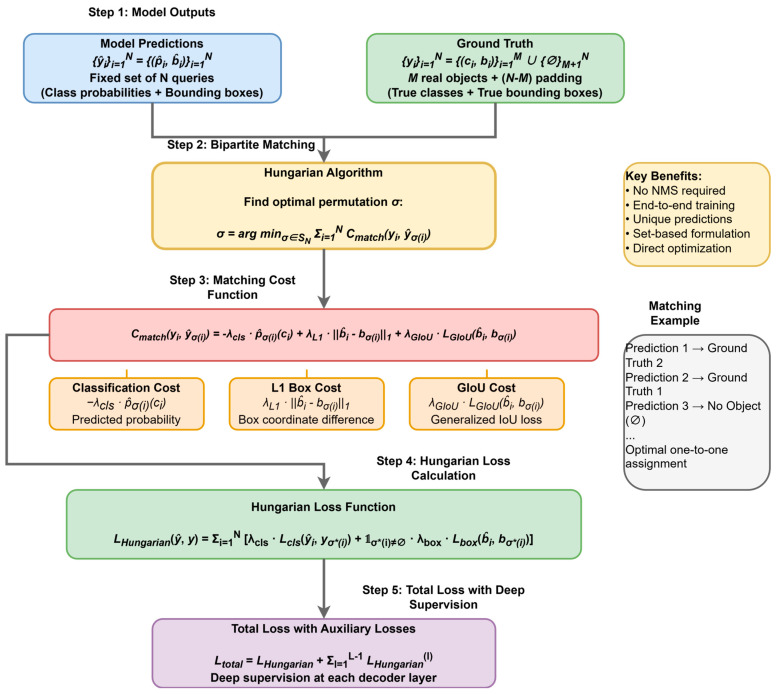
DETR set prediction loss and bipartite matching process flow.

This clearly illustrates the core innovation of DETR—the set prediction loss computation process. Figure 10 presents the complete workflow from model prediction outputs to final loss calculation. This figure provides a detailed description of how DETR achieves optimal matching between predictions and ground truth labels through the Hungarian algorithm, as well as the three key components of the matching cost function: classification cost, L1 bounding box cost, and GIoU cost. Additionally, the figure demonstrates the role of the deep supervision mechanism during the training process.

Here, the cost function is a linear combination of three components, each weighted by a hyperparameter λ:

1. Class Prediction Cost ($-\lambda_{cls}\cdot {\hat{p}}_{\sigma (i)}(c_{j})$): This term scores the classification. Unlike the final loss function, it directly uses the predicted probability ${\hat{p}}_{\sigma (i)}(c_{j})$ of the ground truth class $c_{j}$ to ensure the cost is commensurate with the box regression costs.
2. L1 Bounding Box Cost ($\lambda_{L1}\cdot {\left\|{\hat{b}}_{i}-\left.b_{\sigma (i)}\right\|\right.}_{1}$): This is the L1 distance between the predicted box $\hat{b}$ and the ground truth box b, penalizing differences in center coordinates, height, and width.
3. Generalized IoU Cost ($\lambda_{GIoU}\cdot L_{GIoU}(b_{\sigma (i)},{\hat{b}}_{i})$): This is the Generalized Intersection over Union (GIoU) loss. It is a scale-invariant metric that is more robust than the L1 loss, as it also considers the shape and orientation of the boxes and not just their overlap.

The hyperparameters $\lambda_{cls},\lambda_{L1},\lambda_{GIoU}$ are crucial as they balance the relative importance of these three factors during the bipartite matching process, guiding the Hungarian algorithm to find the optimal one-to-one assignment between predictions and ground truths.

It should be noted that when i=∅, i.e., the object is a background object, its cost is 0, meaning that it does not participate in matching. After finding the optimal matching permutation σ using the Hungarian algorithm, this matching mechanism ensures that each ground truth object is matched by at most one predicted box, thus eliminating the reliance on NMS. However, this also means that the gradient for each predicted box comes only from its uniquely matched ground truth object/background. In the early stages of training, when prediction quality is low, this can lead to unstable matching and sparse supervision issues.

The final Hungarian Loss is defined as the sum of the losses for all matched pairs:

(3) $$L_{H\mathrm{ungarian}}(\hat{y},y)=\sum_{i=1}^{N}\left[\lambda_{cls}\cdot L_{cls}({\hat{y}}_{i},y_{\sigma (i)})+1_{\sigma (i)\neq \emptyset }\cdot \lambda_{box}\cdot L_{box}(b_{\sigma (i)},{\hat{b}}_{i})\right].$$

where $L_{cls}$ is typically the negative log-likelihood loss (NLL Loss) for the K+1 categories, and $L_{box}$ is the bounding box regression loss, which is usually a weighted sum of the L1 loss and the GIoU loss. It should be noted that the bounding box loss is only calculated for predictions matched to a ground truth object (σ(i)≠∅), while the classification loss is calculated for all predictions. This enforced model generates a unique and accurate prediction for each ground truth object and suppresses false positives for the background.

Furthermore, to facilitate model training, DETR connects prediction heads after the output of each decoder layer and calculates the Hungarian loss. These losses from intermediate layers are called Auxiliary Losses. The final total loss is the sum of the losses from all decoder layers (including the last layer). This deep supervision strategy, by introducing supervision sign $L_{box}$ at each decoder layer, provides more direct guidance for the layer-by-layer refinement of object queries, helps alleviate gradient vanishing, and is crucial for stabilizing the training process and accelerating convergence [40].

However, the aforementioned improvements do not come without cost. Bipartite matching can be unstable in the early stages of training due to random matching caused by poor prediction quality, which is one of the main reasons for DETR’s slow convergence [41,42]. Moreover, the model’s performance is also highly sensitive to the weight hyperparameters (λ) of each term in the loss function, requiring meticulous tuning to achieve optimal results [43].

By combining CNN feature extraction, Transformer encoder–decoder, and innovative object queries, DETR frames object detection as an end-to-end set prediction problem. Its core bipartite matching and Hungarian loss mechanism are key to dispensing with anchor boxes and NMS, demonstrating architectural simplicity. However, as previously mentioned, its initial version had significant shortcomings in convergence speed, small object detection, and computational efficiency. These issues have led to the numerous improvements discussed in Section 3: for example, optimizing the attention mechanism (Section 3.1) to enhance the feature representation capability and computational efficiency for objects of different scales, particularly small objects; and improving query design (Section 3.2) and training strategies (Section 3.3) to stabilize the matching process and accelerate convergence, among others.

## 3. Key Challenges and Technical Evolution

**Figure 11:**
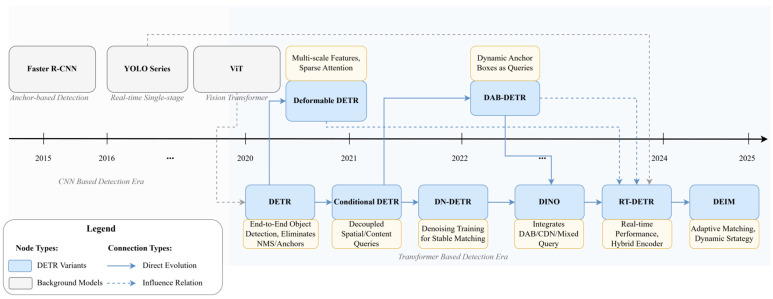
Timeline chart of key DETR variants.

Despite DETR proposing a revolutionary end-to-end object detection framework, its original model exposed three major core challenges in practical applications, slow convergence speed, poor small object detection performance, and low computational efficiency [44,45,46,47], which greatly limited the realization of its potential. These challenges constitute the main driving force behind the subsequent technical evolution of DETR and have catalyzed the rich and diverse variant models discussed in this Section. To overcome these bottlenecks, researchers have made numerous improvements and optimizations to DETR’s architecture and training process from different perspectives, giving rise to a rich and diverse array of DETR variant models. This section will follow a problem-driven perspective, systematically sorting these key variants and technical evolution paths according to the core modules being optimized and deeply analyzing their core innovations, effects, potential limitations, and the inheritance and development relationships between them. Through this approach, to more clearly illustrate the evolutionary path of these key models, Figure 11 first presents a timeline of key DETR variants’ evolution, visually showing the time points of the appearance and mutual influence of these important models. Building upon this, Table 1 further clearly summarizes the main innovations and core problems addressed by several milestone DETR variants that will be discussed in depth in this section, providing an overall context for the subsequent detailed discussion.

This section focuses on the milestone advancements that have successfully addressed DETR’s initial set of problems. By tracing these developments, we aim to build a clear picture of how the community has propelled the DETR architecture to its current level of maturity and highly competitive performance. This analysis of how foundational issues were resolved naturally sets the stage for Section 5, which will explore the persistent frontier challenges that remain.

### 3.1. Enhancing Feature Representation and Efficiency: From Dense to Sparse Attention

Two of the core challenges faced by the original DETR are its low computational efficiency when processing high-resolution feature maps and the resulting poor performance on small object detection. This is primarily due to the fact that the computational complexity and memory consumption of the standard multi-head self-attention (MSA) in its Transformer encoder are proportional to the square of the input sequence’s length. To address these issues, researchers have proposed various schemes to optimize attention calculation efficiency and effectiveness.

**Figure 12:**
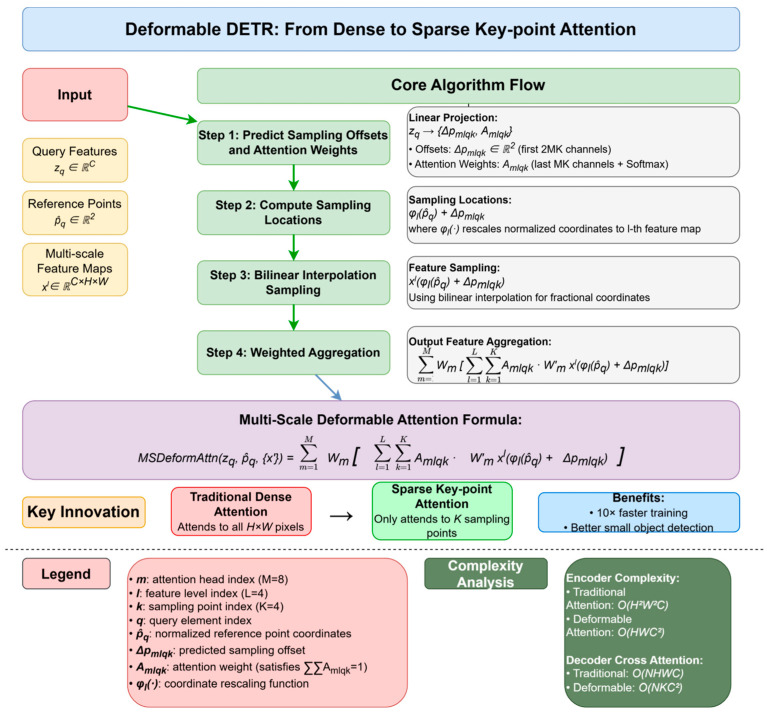
Deformable DETR architecture diagram.

Among these, the deformable attention mechanism proposed by Deformable DETR is one of the most influential improvements, with a schematic diagram shown in Figure 12. Its core idea, inspired by deformable convolution networks [44,45,46,47], is that attention should not be computed over the entire feature map but should instead focus on a small set of key sampling points around a reference point. This paradigm shift from “dense attention” to “sparse key-point attention” marks a critical turning point for DETR’s efficiency and performance. Specifically, given an input query feature $z_{q}$ and its normalized 2D reference point ${\hat{p}}_{q}$, the multi-scale deformable attention module predicts the sampling offset $\Delta P_{mlqk}$ and attention weight $A_{mlqk}$. These are predicted for k sampling points across l feature levels and m attention heads via a linear projection on $z_{q}$. The final output is a weighted aggregation of features sampled from the multi-scale feature map $x^{l}$ at these offset locations using bilinear interpolation. This mechanism drastically reduces the computational complexity. For an encoder layer, it lowers the complexity from $O(H^{2}W^{2}C)$ of traditional self-attention to $O(HWC^{2})$. The effective fusion of multi-scale features and the focus on a sparse set of key points not only significantly improve detection performance, particularly for small objects, but also accelerate training convergence. The success of deformable attention has made it a fundamental component of many subsequent high-performance DETR variants [51]. However, its advantages are also accompanied by some compromises, and the learning of its sampling point locations may not be as stable as global attention, which is particularly evident in the early stages of training. At the same time, only focusing on a few sampling points theoretically may also carry the risk of losing some long-range global contextual information.

Besides the milestone work of deformable attention, other explorations aimed at optimizing attention efficiency and effectiveness have also enriched the technical DETR ecosystem. Below are some examples:

1. Sparse attention reduces complexity by only calculating a portion of important attention weights. Sparse DETR [27] is a representative of this, proposing to only update feature map tokens explicitly referenced by object queries in the decoder, thereby reducing computational costs during backpropagation. Similarly, PnP-DETR [52] optimizes the attention module in a plug-and-play manner, improving flexibility and efficiency. The challenge of these methods lies in how to effectively determine which attention weights are important and worth calculating and how to achieve sparsification without significantly sacrificing performance.
2. Lightweight attention typically combines efficient network structure design. For example, Lite-DETR [53] combines a lightweight network with a sparse attention mechanism, aiming to achieve significant efficiency improvement and latency reduction, which are particularly important for resource-constrained devices but may make certain compromises in accuracy. IA-DETR [54] introduces an indirect attention mechanism, flexibly establishing relationships between object queries, target image features, and query image features; simplifying the traditional cross-attention mechanism; and significantly improving the model’s performance in one-shot object detection.
3. Furthermore, although the Swin Transformer [55] is primarily a general visual backbone network architecture and was not directly applied to the optimization of the DETR’s attention mechanism module, its proposed window-based shifted attention mechanism, which computes attention within local windows and uses window shifting for cross-window information exchange, also provides important insights into how to balance computational efficiency and the receptive field in the Transformer architecture. These diverse attempts collectively promote the development of attention mechanisms within the DETR framework [56].

### 3.2. Stabilizing Training and Accelerating Convergence: Innovations in Query and Supervision

Despite efficient attention mechanisms alleviating some issues, the core bottleneck of the original DETR’s slow convergence, the instability of bipartite matching caused by randomly initialized queries, still persists [57,58]. To fundamentally address this “cold start” problem, researchers are dedicated to improving query design and enhancing training supervision to accelerate convergence and improve accuracy.

**Figure 13:**
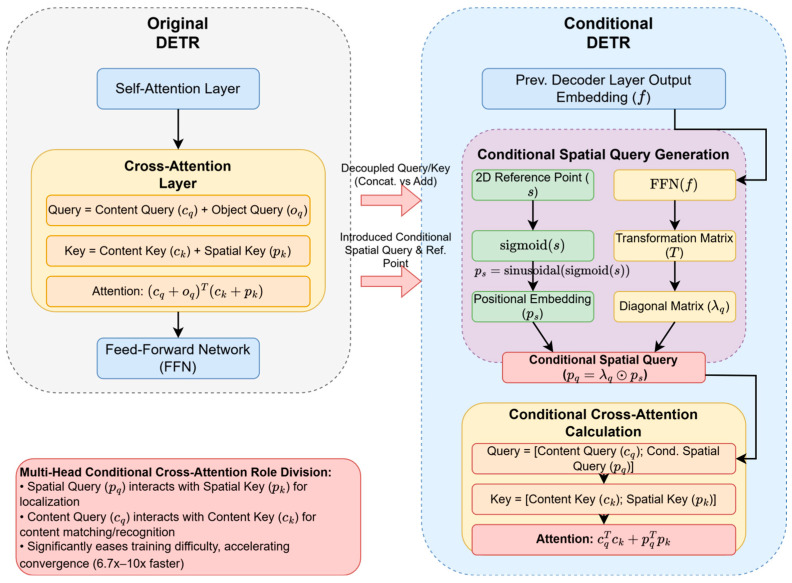
Conditional DETR architecture diagram.

In terms of query design, an important early attempt is conditional DETR [38], which aims to solve the problem of coupling between spatial localization and content recognition in the original cross-attention. Its structure diagram is shown in Figure 13. This study decouples each object query into a content embedding $c_{q}$ and a spatial query (a learnable 2D reference point s). Its core innovation lies in the design of the conditional cross-attention mechanism. Specifically, the reference point s is first normalized through a Sigmoid function, and then, its corresponding positional embedding $p_{s}$ is generated using the same sine/cosine function as the encoder’s positional encoding, as shown in Equation (4).

(4) $$p_{s}=\mathrm{sinusoidal}(\mathrm{sigmoid}(s)).$$

At the same time, the output embedding f from the previous layer of the decoder learns a transformation T through a small FFN (implemented as a diagonal matrix for efficiency, with diagonal element $\lambda_{q}$). This transformation acts on the positional embedding $p_{s}$ of the reference point, generating the conditional spatial query $p_{q}$, and the formula is as follows:

(5) $$p_{q}=Tp_{s}=\lambda_{q}⊙p_{s}$$

When calculating cross-attention, the query is constructed as the concatenation of the content query $c_{q}$ and the conditional spatial query $p_{q}$, and the key is correspondingly constructed as the concatenation of the encoder output content features $c_{k}$ and their positional encoding $p_{k}$. Thus, the computation of attention weights can be decomposed into the sum of the dot products of the content part $c_{q}^{T}c_{k}$ and the spatial part $p_{q}^{T}p_{k}$. Through this design, the spatial query $p_{q}$ is mainly responsible for interacting with the positional encoding $p_{k}$ of the image features to achieve spatial localization, while the content query $c_{q}$ is mainly responsible for interacting with the image content features $c_{k}$ for category recognition. This decoupling greatly reduces the learning difficulty, enabling the model to accelerate and more accurately learn localization and recognition tasks, thereby significantly accelerating convergence (6.7–10 times) and improving detection accuracy.

Building upon the preliminary spatial decoupling introduced by conditional DETR, researchers further explored ways to introduce stronger spatial priors. Anchor DETR [59] directly binds queries to fixed 2D anchor points, providing strong priors and simplifying matching; however, it sacrifices the flexibility of being anchor-free. DAB-DETR [48] goes a step further, parameterizing queries directly as 4D dynamic anchor boxes ($C_{x}$, $C_{y}$, w, and h) and explicitly iterating and optimizing these bounding box parameters layer by layer in the decoder, providing a more flexible and adaptive spatial prior. Other works have also focused on the representation and generation of queries, such as the query reformulation method based on box queries in conditional DETR v2 [60], and the ranking-based adaptive query generation (RAQG) method proposed by Gao et al. [61] to address crowded scenes.

**Figure 14:**
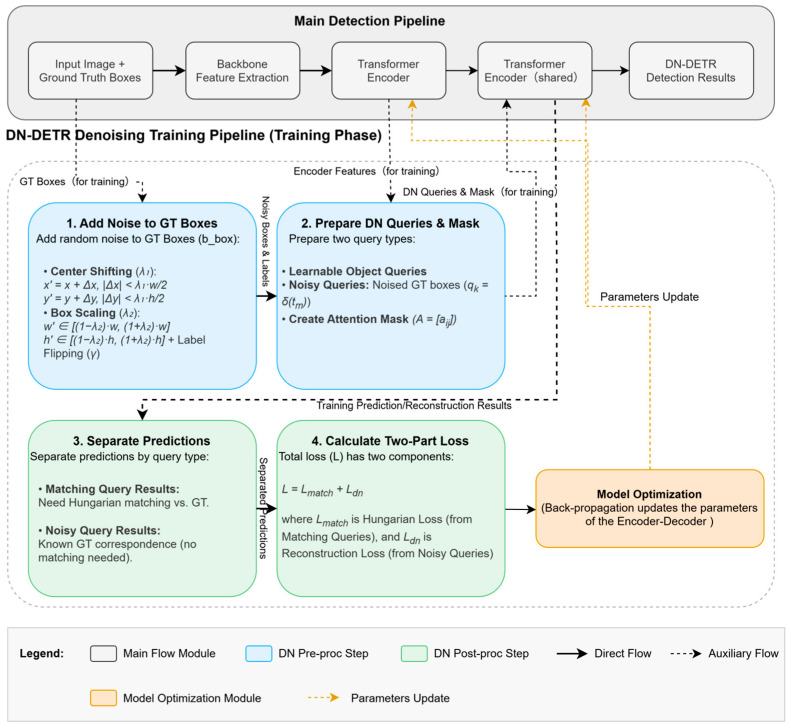
DN-DETR denoising training pipeline (training phase).

In terms of training supervision, the most influential breakthrough is the query denoising training strategy proposed by DN-DETR [28]. The core idea is that in each training iteration, in addition to the original object queries, a batch of “Noisy Queries”—constructed by artificially adding random noise to ground truth (GT) boxes—is also input. The model is then explicitly trained to accurately reconstruct the corresponding noise-free GT boxes from these noisy queries (the pipeline is shown in Figure 14; DN-DETR: denoising training pipeline (training phase)). Because a stable one-to-one correspondence exists between the noisy queries and their corresponding GT objects, this denoising task provides a direct and stable regression supervision signal, effectively bypassing the problem of unstable bipartite matching, which is caused by inaccurate predictions in the early stages of training, thereby greatly stabilizing the training process. The paper claims that, on the COCO dataset, it can achieve comparable or even slightly better performance with only 1/10 of the training epochs of the original DETR (e.g., 2.1 AP higher with 44 M parameters). Today, the query denoising training strategy and its variants have become one of the key technologies for building high-performance DETR models and are widely used in many subsequent works. However, while this strategy of introducing an auxiliary denoising task is effective, it inevitably increases the complexity of the model design and introduces additional hyperparameters (noise type, intensity, etc.) during the training process, posing new challenges for training resource consumption and tuning capabilities.

The idea of combining spatial priors with denoising training led to representative work updated by the DAB-DETR research team—the SOTA model DINO. Building upon preceding work like DAB-DETR and DN-DETR, DINO cleverly integrates multiple advanced technologies, significantly improving model performance relative to SOTA and becoming the first DETR variant model to achieve the top ranking on the COCO object detection benchmark. Compared to previous SOTA detectors, it further reduced model parameters and training data by more than 10 times. The key innovations of DINO include the following: adopting DAB-DETR’s dynamic anchor box detection for query design. In terms of training strategy, DINO builds upon and enhances the denoising training concept from DN-DETR [28] by proposing contrastive denoising training (CDN). As detailed in Algorithm 1 of Table 2, CDN significantly improves effectiveness by introducing two types of noised queries derived from each ground truth (GT) box: positive queries (GT boxes with small noise, tasked with reconstructing the original GT) and negative queries (GT boxes with larger, yet still correlated, noise, tasked with being classified as “no object”). This contrastive approach compels the model to more precisely learn the boundary between true objects and very similar negative examples, thereby reducing duplicate detections and improving localization accuracy.

Furthermore, DINO introduces mixed query selection (detailed in Algorithm 2 of Table 2). This mechanism distinctively initializes the decoder queries: The positional queries (serving as initial anchor box proposals) are dynamically derived from the spatial information of the Top-K most salient features selected from the encoder’s output, providing image-adaptive spatial priors. Concurrently, the content queries remain as independent learnable embeddings, allowing the decoder to focus on object appearance features without being overly biased by the initial content of selected encoder features. This hybrid initialization of queries, combining content-agnostic learnable embeddings with content-aware positional priors, contributes to more stable and efficient training. DINO also incorporates an improved box optimization mechanism known as “Look Forward Twice”. The core algorithmic principles of DINO’s contrastive denoising and mixed query selection are exemplified in Table 2.

**Table 2:** DINO core algorithm step example.

**Algorithm Name:** Core Algorithm for Contrastive Denoising and Hybrid Query Selection
Algorithm 1. Contrastive Denoising (CDN) Training Method
**Input:** Image, gt_boxes(Ground Truth Bounding Box), gt_labels(Ground Truth Labels)
**Output:** Total Training Loss
function Contrastive_Denoising_Training(image, gt_boxes, gt_labels):
Algorithm 2. Mixed Query Selection
**Input:** Encoder_features: Output features from the Transformer Encoder
**Output:** Initial_Decoder_Queries: Initial queries for the Decoder {positional_queries, content_queries}
Function Mixed_Query_Selection (Encoder_features):
Helper Function Stubs (Conceptual): Generate_CDN_Query_Groups(…): encapsulates creating positive (target: GT) and negative (target: no-object) samples; Initialize_Matching_Queries(…): returns initial anchors and content for the matching part (could use Mixed Query Selection for anchors); Select_Top_K_Feature_Locations(…): identifies promising regions from Encoder features; Generate_Anchor_From_Locations(…): derives initial anchor boxes from these location.

In addition, optimizing the matching and loss functions also serves as an important supplement to accelerate convergence. DEIM [50] proposed an innovative training framework that introduces a dense O2O matching strategy and matchability-aware loss (MAL) to increase positive sample density and improve match quality. SAM (semantic-aligned matching) [62] introduced semantic alignment information when calculating matching costs to make the matching process more stable. Additionally, some works have also optimized at the loss function level, for example, by introducing focal loss [63] to address the positive–negative sample imbalance or using more advanced losses like GIoU [64] and DIoU/CIoU [65] to provide more effective regression gradients. Rank-DETR [66] proposed a ranking-based loss function, aiming to directly optimize the AP evaluation metric. To handle crowded scenes, recurrent DETR [67] introduced Pondering Hungarian Loss, while Align-DETR [40] proposed Align Loss to address the inconsistency between the classification score and localization accuracy. These targeted loss function designs have all contributed to improving DETR’s performance and robustness in specific scenarios [68,69].

Beyond the algorithmic innovations discussed above, successful DETR training also relies on the careful configuration of key strategies. Understanding these configurations is crucial for practitioners aiming to apply DETR:

1. Loss Weight Configuration: The total loss in DETR is a weighted sum of the classification loss, L1 loss, and GIoU loss. The configuration of these weight hyperparameters ($\lambda_{cls},\lambda_{L1},\lambda_{GIoU}$) is critical [43]. In the original DETR, they were set to (1, 5, 2) to balance the gradient scales of different tasks. In practice, if the model performs poorly in localization, one can try to increase the weights of $\lambda_{L1}$ and $\lambda_{GIoU}$; conversely, if classification errors are frequent, the weight of $\lambda_{cls}$ can be increased.
2. Learning Rate Scheduling: DETR training is very sensitive to the learning rate schedule. A common effective practice is to set different learning rates for the CNN backbone and the Transformer module. The backbone, typically using pre-trained weights, requires a smaller learning rate (e.g., 10× smaller than the Transformer part) for fine-tuning. Additionally, a short “warmup” phase at the beginning of training is crucial for stability [70].
3. Data Augmentation Strategies: Standard data augmentation methods like random flipping, scaling, and cropping are effective for DETR. It is important to adjust the bounding box and positional encoding coordinates accordingly when resizing images. For models aiming to improve convergence speed or handle dense scenes (e.g., DEIM), advanced techniques like Mosaic and Mixup can be considered.

### 3.3. Achieving Real-Time Detection: Architectural Optimization and Specialization

Although early improvements like Deformable DETR improved efficiency to some extent, the computational and memory costs of the original DETR and its high-performance variants are still too high for many real-time application scenarios (e.g., requiring >30 FPS). Therefore, how to significantly optimize the DETR architecture to achieve real-time inference while maintaining high accuracy has gradually become an extremely important research direction. The core challenge in this direction lies in the inherent quadratic complexity of the Transformer model and the accumulated computation brought by the multi-layer encoder–decoder in the DETR model.

**Figure 15:**
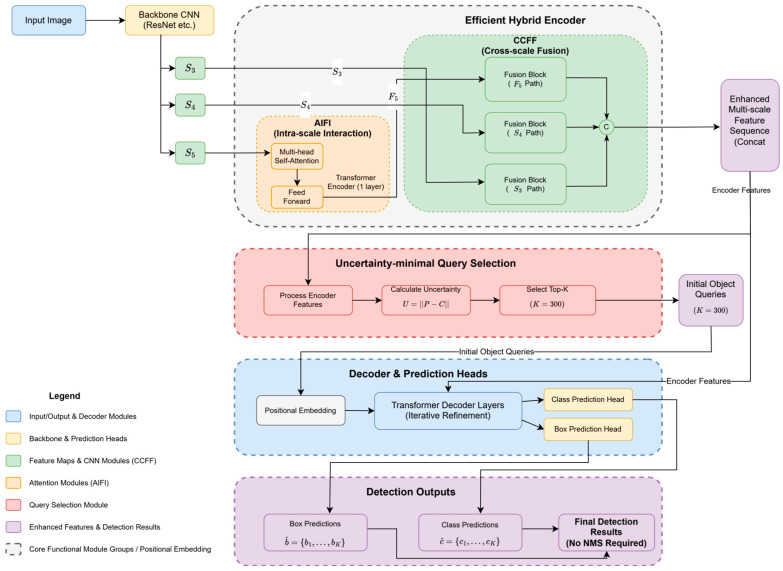
RT-DETR network architecture diagram.

RT-DETR [49] marks a significant advancement in real-time object detection. Instead of merely compressing existing DETR models, it introduces a novel and efficient architectural framework, a schematic of which is depicted in Figure 15. A key innovation is its efficient hybrid encoder. This encoder uniquely combines CNN-based modules for efficient multi-scale feature processing with attention mechanisms for capturing global context. Specifically, the backbone CNN (e.g., ResNet) extracts multi-scale feature maps, denoted in Figure 15 as “S3”, “S4”, and “S5”. These correspond to outputs from different stages of the backbone: “S3” from an earlier stage, offering high resolution and fine-grained details with shallower semantic information; “S4” from an intermediate stage, balancing resolution and semantics; and “S5” from a deeper stage, providing rich semantic information and abstract features at a lower resolution. These multi-scale features (S3, S4, and S5) are inputs to the Efficient Hybrid Encoder.

The Efficient Hybrid Encoder is central to RT-DETR’s design and comprises two main sub-modules: the attention-based intra-scale feature interaction (AIFI) module and the CNN-based cross-scale feature fusion (CCFF) module.

1. AIFI Module: as illustrated, the AIFI module specifically processes the highest-level feature “S5”. It employs a single Transformer encoder layer, which internally consists of multi-head self-attention followed by feed-forward operations. This allows “S5” features to undergo intra-scale interaction, effectively capturing global contextual dependencies within that scale and producing enhanced features denoted as “F5”.
2. CCFF Module: The CCFF module is responsible for integrating features across different scales. It receives the AIFI-enhanced “F5” features along with the original “S3” and “S4” features from the backbone. Within the CCFF block shown in Figure 15, these multi-scale features (F5, S4, and S3) are channeled into distinct parallel “Fusion” paths (labeled as “Fusion (F5 Path)”, “Fusion (S4 Path)”, and “Fusion (S3 Path)”). These paths facilitate bi-directional information flow (indicated by dashed arrows in the diagram, representing top-down and bottom-up interactions similar to a Path Aggregation Network structure), allowing features from different levels to be effectively fused. The outputs resulting from these interactive fusion paths are then aggregated, typically via concatenation (symbolized by “C” in the original RT-DETR paper’s conceptual diagram and implied by the merging arrows in Figure 15), to form the final enhanced multi-scale feature sequence. This enhanced multi-scale feature sequence serves a dual purpose: it is flattened and then passed to the uncertainty-minimal query selection module, and simultaneously, it is directly provided as encoder features to the subsequent decoder and prediction heads.
3. Uncertainty-Minimal Query Selection: This module takes the flattened enhanced multi-scale feature sequence from the CCFF. Internally, it processes these encoder features, calculates an uncertainty metric (e.g., based on the discrepancy between predicted localization P and classification C distributions, U = ∣∣P − C∣∣) and then selects the Top-K (e.g., K = 300) features. These selected features, exhibiting minimal uncertainty and thus representing high-quality candidates with strong joint localization and classification confidence, are used to form the “Initial Object Queries”. This selective mechanism significantly reduces the number of queries that proceed to the computationally intensive decoder stage.
4. Decoder and Prediction Heads: The decoder block receives two primary inputs: the “Initial Object Queries”(K = 300) and the complete “Enhanced Multi-scale Feature Sequence” (as “Encoder Features”). The object queries are first combined with “Positional Embedding” to incorporate spatial information. They are then iteratively refined through multiple “Transformer Decoder Layers”, attending to the encoder features. Finally, the refined queries from the decoder are fed into a separate “Class Prediction Head” and “Box Prediction Head” to generate the “Class Predictions” ($\hat{c}$) and “Box Predictions” ($\hat{b}$), respectively.
5. Detection Outputs: The outputs from the prediction heads constitute the “Decoder Outputs”. Notably, RT-DETR produces the “Final Detection Results” without requiring Non-Maximum Suppression (NMS), which is a significant advantage for real-time performance.

These architectural innovations enable RT-DETR to achieve inference speeds significantly surpassing previous DETR variants while maintaining detection accuracy comparable to, or even exceeding, the YOLO series. This powerfully demonstrates the DETR architecture’s potential in real-time detection tasks.

To comprehensively and quantitatively evaluate the performance and efficiency of mainstream DETR models, we conducted a series of rigorous tests on the COCO dataset and summarized the detailed comparison results in Table 3. We adopted multi-dimensional evaluation metrics widely recognized in the object detection field, which can be divided into two main categories: model performance and model efficiency:

1. Performance Metrics: The evaluation of performance focuses not only on whether the model can detect objects but also on the accuracy of its localization and its robustness across different scenarios.
  - AP (Average Precision): This is the core evaluation metric for the object detection task. It integrates the model’s precision and recall, calculated as the area under the precision–recall (P-R) curve across different confidence thresholds. In the context of the COCO dataset, AP typically refers to the average of AP values calculated at 10 different IoU thresholds, ranging from 0.5 to 0.95 with a step of 0.05. This comprehensively reflects the model’s overall performance under various localization accuracy requirements.
  - AP50 and AP75: These are AP values at specific IoU thresholds. AP50 (AP at IoU = 0.50) uses a relatively lenient IoU criterion (0.5), a traditional metric from the PASCAL VOC challenge, which primarily measures the model’s ability to detect objects. In contrast, AP75 (AP at IoU = 0.75) employs a stricter localization standard, requiring a higher degree of overlap between the predicted and ground truth boxes, thereby better reflecting the model’s precise localization capability.
  - APs, APm, and APl: This set of metrics is used to evaluate the model’s performance on objects of different scales, which is crucial for analyzing its scale robustness. According to the COCO definition, APs correspond to small objects (area < 32 × 32 pixels), APm to medium objects (32 × 32 < area < 96 × 96 pixels), and APl to large objects (area > 96 × 96 pixels). This data allows for an analysis of whether the model has weaknesses in detecting objects of specific sizes, particularly small ones.
2. Efficiency Metrics: In addition to performance, the model’s computational cost and inference speed are key to measuring its practical value.
  - Parameters: These refer to the total number of learnable parameters in the model, usually measured in millions (Ms). It directly determines the model’s size, affecting storage requirements and loading times.
  - GFLOPs (Giga Floating-Point Operations): This indicates the number of Giga Floating-Point Operations required for a single forward pass of the model. It is a theoretical metric for computational complexity, decoupled from specific hardware, allowing for a fair comparison of the computational demands of different models.
  - FPS (Frames Per Second): This measures the number of image frames that the model can process per second on specific hardware. It is a highly practical metric that directly reflects the model’s operational speed in real-world deployment scenarios.

Based on these metrics, we present a comprehensive comparison of key DETR variants and established real-time detectors in Table 3.

A thorough analysis of the results in Table 3 reveals several key insights. First, when compared to its DETR predecessor, RT-DETR fundamentally breaks the established trade-off where accuracy gains necessitate a drop in speed. High-performance variants like Sparse DETR, while achieving a strong 49.3 AP, operate at only 17.2 FPS. In contrast, RT-DETR-R50 (ResNet-50) not only surpasses this accuracy with an impressive 53.1 AP but also achieves a remarkable 108 FPS, demonstrating a simultaneous and significant improvement in both domains.

Second, and more critically, RT-DETR establishes itself as a top-tier competitor against the YOLO series, the long-standing benchmark for real–time detection. The comparison highlights two crucial points:

3. At a similar level of performance: RT-DETR-R50 (53.1 AP) achieves comparable accuracy to the SOTA YOLOv8l (52.9 AP) and YOLOv10l (53.2 AP) while maintaining a highly competitive inference speed of 108 FPS.
4. At a similar speed level: When comparing models in the ~100 FPS range, RT-DETR-R50’s accuracy of 53.1 AP is significantly higher than that of established models like YOLOv5l (49.0 AP).

This robust performance powerfully demonstrates that the DETR architecture, through the innovations in RT-DETR, is no longer confined to high-accuracy, low-speed scenarios but has become a potent and flexible framework for real-time detection tasks.

Nevertheless, these advantages are accompanied by certain trade-offs. Compared to the pure CNN architecture of the YOLO series, RT-DETR’s hybrid encoder is architecturally more complex, which can pose greater challenges for deployment and optimization. Furthermore, its efficiency-driven query selection mechanism might risk overlooking some valid targets, particularly in scenarios with dense object distributions or numerous small objects.

In addition to RT-DETR, other research directions aimed at improving efficiency mainly include the following four types:

1. Architecture Simplification and Improvement: D^2^ETR [77] explored the possibility of using only the decoder. Recurrent DETR attempts to introduce a recursive mechanism to process temporal data to improve real-time performance. RT-DETRv2 [78] further enhances the practicality and performance of RT-DETR by optimizing the training strategy and introducing adjustments that do not increase inference cost (Bag of Freebies).
2. Model Compression: Model quantization [79] aims to reduce the number of parameters and computations of the model by reducing the bit width of model parameters and activation values to decrease model size and accelerate computation. AQ-DETR [80] explored low-bit quantization-aware training for DETR. Pruning [81] reduces complexity by removing redundant parameters or structural units (such as attention heads, FFN units, etc.) from the model. Pruning DETR [82] improved the inference efficiency of the model through a sparse structured pruning method.
3. Lightweight Design: These methods directly start from the architectural level, aiming to design lighter DETR variant models. This includes adopting sparse attention mechanisms (e.g., research like Deformable DETR, LITE-DETR, etc.), using lightweight CNN backbone networks including MobileNet [83], and reducing the number of layers or hidden dimensions of the Transformer encoder–decoder. For example, L-DETR [84] balances the efficiency and accuracy of object detection by combining the DETR framework with the lightweight backbone network PP-LCNet [85].
4. Edge-Side Optimization: With the growing demand for edge computing, research efforts have begun to focus on combining hardware characteristics and algorithm optimization to efficiently deploy DETR models on resource-constrained edge devices. Works like SpeedDETR [86] introduced hardware-aware latency prediction models to guide Transformer architecture design, achieving efficient inference on edge GPUs while balancing accuracy and speed. This direction requires closer collaboration between algorithm designers and hardware engineers.

### 3.4. Specialized Functionality Expansion: Broadening Application Boundaries

DETR’s unique end-to-end framework, query-based mechanism, and set prediction characteristics have not only revolutionized the object detection paradigm but also laid the foundation for its application in a wider range of visual tasks. Researchers have begun to attempt and successfully extend the core ideas of DETR to various visual perception domains, demonstrating the potential of this architectural paradigm in solving broader visual problems [87]. This unified detection and segmentation framework avoids the complex RoIAlign operation in traditional two-stage instance segmentation methods, making the process simpler.

#### 3.4.1. Dense Prediction Tasks

An important direction of expansion is Dense Prediction Tasks, such as instance segmentation [88] and panoptic segmentation [89]:

1. Instance Segmentation: Since DETR essentially performs set prediction, it can be naturally extended to simultaneously predict bounding boxes and pixel-level masks for objects. For example, Mask DINO [90], based on DINO, achieved leading performance in instance segmentation tasks by adding a parallel mask detection head and combining strategies such as instance-level contrastive learning.
2. Panoptic Segmentation: DETR’s set prediction idea is also applicable to panoptic segmentation, a task that requires simultaneously segmenting and identifying “Things” and “Stuff” in an image. The original DETR paper has already initially verified its feasibility for this task. Subsequent research, such as Panoptic SegFormer [19], further optimized its panoptic segmentation capabilities by combining a DETR-style set prediction mechanism with a semantically guided segmentation head, improving the consistency of the model at the semantic and instance levels, and significantly increasing panoptic segmentation accuracy, especially in modeling “Stuff” regions.

#### 3.4.2. Three-Dimensional (3D) Vision Tasks

3D vision is another important direction for the application of DETR’s ideas. Extending DETR from 2D images to 3D space faces challenges in data representation and computational complexity. BEVFormer is a representative work for handling 3D detection and tracking in autonomous driving scenarios. It innovatively utilizes a spatiotemporal Transformer to efficiently aggregate temporal image features from multiple cameras into the Bird’s-Eye-View (BEV [91]) space and then applies a DETR-like query mechanism on the unified BEV representation for 3D object detection. PETR [92] applies DETR’s query mechanism to multi-view 3D object detection, incorporating 3D coordinate information into queries through positional embedding transformation and enabling them to directly interact with 2D image features. These works indicate that DETR’s query paradigm can effectively handle complex 3D spatial relationships and multi-view information fusion, providing new solutions for 3D perception.

#### 3.4.3. Open-Vocabulary Object Detection Tasks

Open-Vocabulary Object Detection (OVD) [93] aims to overcome the limitation of traditional detectors that can only recognize a predefined set of categories, empowering models to detect new categories specified by arbitrary text descriptions [94]. The DETR architecture, particularly its decoupled query mechanism and independent classification prediction head, provides a natural advantage for achieving Open-Vocabulary Object detection. Researchers have effectively leveraged the rich semantic knowledge embedded in large-scale Vision-Language Models (VLMs), such as CLIP [95], by devising methods to effectively integrate this knowledge into the DETR framework for Open-Vocabulary Detection (OVD). A common strategy involves several key steps: First, the text encoder component of a VLM is utilized to generate high-dimensional text embeddings for arbitrary, user-defined class names. These text embeddings capture the semantic essence of the categories. Second, these dynamically generated text embeddings are then employed to augment or replace the conventional learned parameters within the DETR classification head. For instance, instead of fixed classifier weights learned for a predefined set of categories, these text embeddings can serve as adaptable “prototypes” or target vectors for object classes. Third, during the training phase, a critical alignment between the image region features (produced by the DETR decoder) and their corresponding text embeddings is typically enforced using a contrastive loss function. The contrastive loss works by pulling the feature representations of an image region and its correct textual description closer together in the embedding space, while simultaneously pushing apart the representations of the image region and incorrect or irrelevant textual descriptions. Through this explicit training objective, the model learns to establish a robust association between visual features and the semantic concepts encapsulated by the text embeddings. Consequently, during the inference phase, the model can identify and localize objects belonging to categories not encountered during training. This is achieved by simply providing the novel class names, converting them into text embeddings using the same VLM text encoder, and then matching these new embeddings against the image region features [96]. The successful implementation of OVD through this mechanism significantly enhances the practicality and generalization capabilities of DETR, enabling these models to be deployed in broader and more dynamic application scenarios where the set of target objects is not known beforehand.

#### 3.4.4. Other Frontier Vision Tasks

Furthermore, the DETR framework has also been widely applied in other vision tasks:

1. Video Understanding: Including video object detection and tracking, which can maintain object identity by associating object queries across frames [97] or introduce recursive structures to process temporal information [98].
2. Continual/Incremental Learning: Research on how to prevent DETR models from forgetting old categories when learning new ones to better address the challenges of expensive data annotation or continuously increasing categories in the real world. Works such as Incremental-DETR [99] and Continual Detection Transformer [100] have studied how to effectively mitigate catastrophic forgetting in DETR models when learning new categories.
3. Weakly/Semi-Supervised Learning: Dedicated to completing tasks with limited data annotation. For example, works like Semi-DETR [101] have explored training paradigms that combine a large amount of unlabeled data.
4. Uncertainty Estimation: For safety-critical application areas such as autonomous driving, predicting uncertainty is crucial. Some works, including E-DETR [102], have attempted to introduce theories like Evidence Deep Learning [103] into DETR to quantify prediction confidence.
5. Domain Adaptation: The goal is to improve the generalization ability of models across different environments and datasets. Works represented by Mean Teacher DETR [104] utilize consistency regularization to align predictions between source and target domains.
6. Scene-Specific Optimization: Some works also need to specifically consider object characteristics or environmental factors in specific scenes and optimize accordingly. Examples include S-DETR [105] for marine vessel detection and improved RT-DETR [106] focused on robot vision.
7. Hybrid Models and Automation: To further improve performance and reduce design costs, hybrid models [107] that combine the advantages of DETR with other detectors (such as YOLO), as well as automated DETR design using techniques like Neural Architecture Search, are also emerging research directions [108].
8. Multimodal Fusion: Multimodal perception tasks fuse various types of information, such as images, text, and point clouds, to complete more complex perception tasks. DETR has shown great potential in such task scenarios [109,110,111].

In summary, the core design philosophy of DETR has proven to be highly extensible, and its application boundaries are continuously being broadened to various subfields of CV. This not only validates DETR’s success as an object detector but also reveals its immense potential as a general visual perception and reasoning framework.

In Section 3, from a “problem-driven” perspective, we have delved into the key technical evolutions spurred by DETR’s initial challenges. These advancements have not only led to innovations in model architecture but have also introduced new hyperparameters that are critical to model performance and convergence speed. To provide researchers and practitioners with a clear and practical guide for tuning, we conclude this section by systematically summarizing the key sensitive hyperparameters and their significance for the milestone DETR variants discussed in Table 4.

## 4. Applications of DETR in Specific Domains

**Figure 16:**
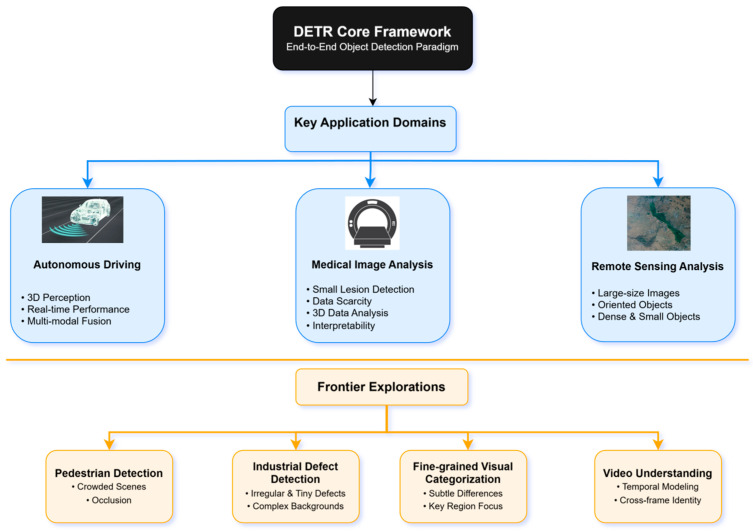
Application ecosystem of the DETR framework.

The end-to-end object detection paradigm based on Transformer, pioneered by DETR, has achieved significant progress not only on generic object detection benchmarks but has also inspired researchers to apply its core ideas to solve visual perception problems in specific domains, leveraging its powerful global context modeling capabilities and the simplicity of discarding hand-crafted components. Different application domains often present unique challenges, such as the extreme demands for real-time performance, robustness, and 3D spatial understanding in autonomous driving [112]; the focus on small object detection accuracy, data scarcity, and interpretability in medical image analysis [113]; and the requirements for large-size input, dense small objects, and rotation invariance in remote sensing image processing [114]. This section aims to systematically review the adaptation and application of DETR and its variants in autonomous driving, medical image analysis, remote sensing image analysis, and several other representative domains. We will delve into how researchers have made adaptive modifications and extensions to the DETR architecture based on domain characteristics, analyze the advantages and limitations demonstrated in solving practical problems, and thereby argue for DETR’s general potential as a flexible and powerful visual perception framework. To provide a more intuitive overview of the application landscape of DETR across different visual perception domains and its technical adaptation roadmap, Figure 16 systematically summarizes the key application domains and frontier explorations covered in this section, along with the core challenges faced in each area.

### 4.1. Autonomous Driving

Autonomous driving systems place extremely high demands on the accuracy, robustness, and real-time performance of environmental perception [115], making it one of the most complex and challenging application scenarios in the CV field. The core lies in accurately understanding the dynamically changing traffic environment, where object detection and tracking play a foundational role in ensuring safety. The specific challenges in this scenario mainly arise from the following:

1. The wide variety of traffic participants, such as vehicles, pedestrians, cyclists, and traffic lights, and their complex and dynamic behavior patterns.
2. Severe challenges to the robustness of perception algorithms posed by drastic changes in environmental factors such as lighting and weather.
3. Frequent mutual occlusion phenomena between objects.
4. High demand for accurate estimation of objects’ precise position, size, and pose in 3D space.
5. The need to meet the stringent computational efficiency and low latency required for real-time vehicle decision-making [116].

DETR-like models, particularly variants optimized for efficiency and multi-scale feature processing, offer new ideas for addressing these challenges due to their end-to-end design philosophy and powerful context modeling capabilities [44].

#### 4.1.1. Three-Dimensional Spatial Perception: A Vision-Based Paradigm Shift

Accurate 3D spatial perception is a prerequisite for safe autonomous driving. Traditional 3D object detection has heavily relied on LiDAR for its precise depth information. However, the high cost and sparse point clouds of LiDAR limit its large-scale application and detailed object understanding. Consequently, low-cost, camera-only 3D detection solutions have become a research hotspot. Compared to traditional monocular or stereo vision-based methods that require complex geometric post-processing, DETR-based multi-view approaches offer a more elegant, end-to-end path.

**Figure 17:**
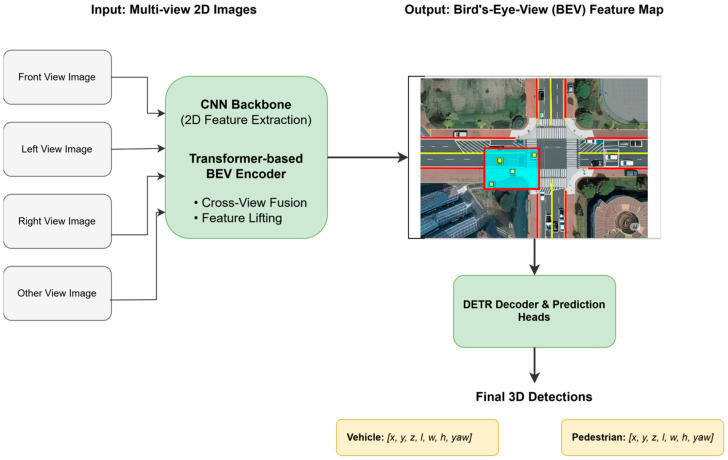
Workflow for generating a BEV representation for 3D object detection.

DETR3D [117] is a representative work in this direction. It proposed an innovative query mechanism that directly establishes the connection between 3D spatial queries and 2D image features by projecting 3D reference points back onto the 2D image plane for feature sampling, avoiding reliance on dense 3D feature representations (like point clouds or voxels). The subsequent BEVformer went a step further, leveraging a spatiotemporal Transformer to efficiently fuse multi-camera, multi-frame information into a unified Bird’s-Eye-View (BEV [91]) representation. The typical workflow for generating such a BEV representation is illustrated in Figure 17. This process generally begins by extracting features from multiple 2D camera views, followed by a core Transformer-based module that fuses these features and “lifts” them into a unified 3D BEV space, where object detection is ultimately performed.

The advantage of this DETR-driven paradigm lies in its ability to simplify the complex “2D detection→ depth estimation → 3D box fitting” pipeline of traditional vision-based 3D detection in an end-to-end manner while utilizing low-cost cameras to perceive rich texture and color information. However, this vision-only approach also has inherent limitations. Compared to the direct distance measurement of LiDAR, its learned depth estimation accuracy is relatively lower, leading to larger localization errors for distant or small objects, and its performance significantly degrades in adverse weather or lighting conditions. Nevertheless, the BEV paradigm has become mainstream due to its friendliness to downstream planning and control modules, and DETR’s query mechanism is the core technical support for realizing this paradigm.

#### 4.1.2. Robustness Enhancement: New Avenues for Multi-Model Fusion

To enhance perception robustness, multi-modal fusion has become a key technology. Traditional fusion strategies, such as simply projecting LiDAR points onto images for feature concatenation (early fusion) or fusing detection results at the decision level (late fusion), often fail to fully exploit the complementary information across modalities. DETR-based BEV fusion methods offer a superior solution. For example, works like BEVFusion [118] and PLC-Fusion [119] first extract features from LiDAR point clouds and multi-view images and transform them into a unified BEV space. They then design dedicated fusion layers (often based on attention) to deeply combine the BEV features of these two modalities. The comparative advantage over traditional methods lies in the following: The Transformer’s cross-attention mechanism can globally model the complex correlations between sparse geometric point cloud features and dense image texture features, achieving a deeper and more effective feature alignment and complementation than simple concatenation or projection. However, how to design DETR variants that can fully utilize multi-modal fused features and maintain the end-to-end property during the fusion process remains an important challenge that needs attention.

#### 4.1.3. Real-Time Performance and Efficiency: A Head-to-Head with CNNs

Real-time performance is a hard metric for deploying any perception algorithm on autonomous vehicles. The original DETR and its early variants struggled to meet real-time processing demands due to high computational complexity. The emergence of models like RT-DETR [49] marked a huge breakthrough in efficiency for the DETR architecture. Compared to traditional real-time detectors (e.g., the YOLO series), the advantage of RT-DETR is its ability to provide higher detection accuracy (AP) at similar inference speeds (e.g., in the ~100 FPS range), thanks to its hybrid encoder retaining the Transformer’s global context modeling capability. Nevertheless, this performance advantage comes with increased architectural complexity. RT-DETR’s hybrid encoder and query selection mechanism are more complex than the pure CNN architecture of YOLO, which may pose more challenges for deployment and optimization, and its ecosystem is far less mature than that of the YOLO series. Therefore, combining general efficiency optimization techniques with the specific requirements of autonomous driving and conducting targeted adaptation and evaluation on relevant benchmark datasets (such as NuScenes [120] and KITTI [121]) remains a key challenge in achieving low-latency, low-power deployment that meets automotive-grade requirements. Simultaneously, hardware–algorithm co-design, which considers the characteristics of the target hardware platform during the algorithm design stage, is also crucial for achieving ultimate performance optimization [122].

### 4.2. Medical Image Analysis

Medical imaging is a crucial basis for clinical diagnosis and treatment decisions. Utilizing computer vision technology for automated analysis, especially achieving high-precision lesion detection and segmentation, is essential for improving diagnosis and treatment efficiency and accuracy [123]. However, the field of medical image analysis faces a series of unique challenges, including diverse lesion shapes and blurred boundaries [124], scarcity and the high cost of high-quality annotated data [125], difficulty in detecting early tiny lesions [126], the need for 3D data processing [127], and strict requirements for high reliability and interpretability [128]. The end-to-end framework and global modeling capabilities provided by DETR and its variants offer new opportunities to address these challenges.

#### 4.2.1. Advantages and Comparison in Dense and Small Lesion Detection

**Figure 18:**
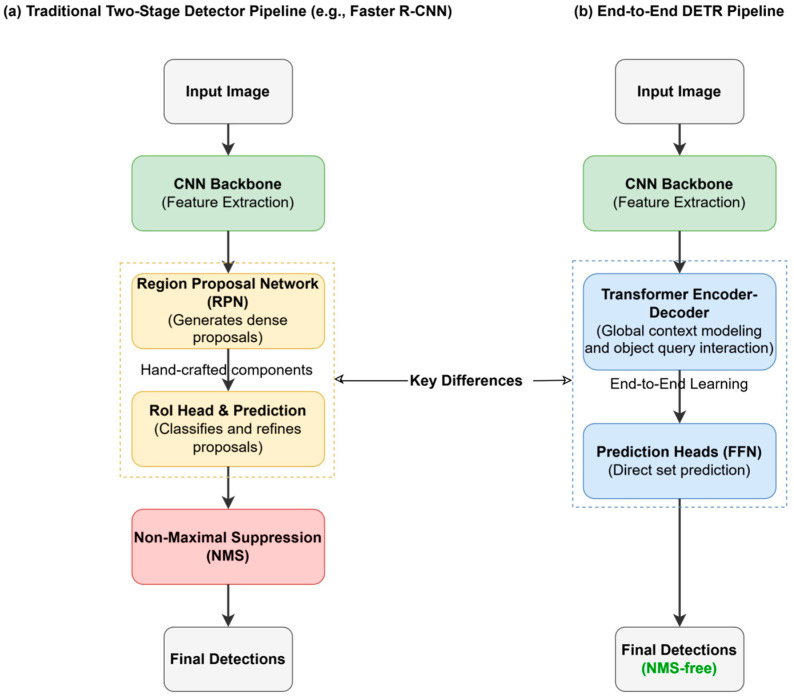
Comparison of object detection pipeline: the multi-stage traditional approach versus the end-to-end DETR framework.

For multi-lesion and dense lesion scenarios in medical imaging, DETR’s end-to-end set prediction paradigm shows significant advantages over traditional methods. Conventional segmentation modes (e.g., U-Net) or detection models (e.g., Faster R-CNN) often require complex post-processing steps like connected-component analysis or NMS to output multiple instances. When lesions are dense or in contact, NMS can easily mistake two separate lesions for overlapping predictions of the same target and suppress one, leading to false negatives. In contrast, DETR’s Hungarian matching mechanism assigns a unique ground truth target to each prediction, fundamentally and thus theoretically avoiding NMS. To clearly illustrate this fundamental difference, Figure 18 provides a visual comparison between the multi-stage pipeline of a traditional detector, which relies on multiple hand-crafted components such as an RPN and NMS, and the more streamlined, end-to-end learning paradigm adopted by DETR. At the same time, the Transformer architecture’s global context modeling capability helps the model understand the complex relationships between lesions and surrounding anatomical structures (such as organs and blood vessels), thereby more accurately distinguishing between lesions and normal tissue and showing great potential, especially when dealing with lesions with blurred boundaries, low contrast, or irregular shapes [129]. For the common challenge of small lesion detection in medical images, improved DETR variants provide effective solutions. For example, by introducing multi-scale feature fusion or designing query mechanisms specifically for small objects, the model’s sensitivity to tiny lesions can be improved. Some research has analyzed the differences in DETR’s application in natural images and medical images, simplifying the DETR model for small lesions in mammograms to significantly improve its performance in small lesion detection tasks [130]. However, detecting extremely small lesions with very low signal-to-noise ratio remains a significant challenge, requiring more refined feature representations and more robust detection strategies.

#### 4.2.2. Addressing Data Scarcity and Class Imbalance

Data scarcity is a relevant bottleneck in medical image analysis. As a model that typically requires large-scale data pretraining, DETR faces severe challenges. Combining efficient learning strategies is key to overcoming this. Unlike traditional methods that rely on complex, modality-specific (e.g., CT, MRI) data augmentation techniques, DETR-based solutions tend to leverage self-supervised learning [131] for pretraining on large amounts of unlabeled medical images, enabling the model to learn domain-relevant general visual features. Furthermore, combining DETR with semi-supervised learning frameworks, as demonstrated by Semi-DETR [101], which trains the model using a small amount of labeled data and a large amount of unlabeled data, is also an effective way to alleviate data scarcity.

Class imbalance (e.g., far more normal tissue pixels than lesion pixels) is another common problem. Traditional methods typically address this at the loss function level, for instance, using Dice Loss in segmentation tasks or Focal Loss [63] in detection. DETR and its variants have also adopted these mature loss functions. However, DETR’s end-to-end nature provides an additional advantage: since there is no interference from an RPN stage, the class imbalance problem can be optimized more directly in the final set-based loss, theoretically allowing for the more effective handling of rare classes.

#### 4.2.3. Three-Dimensional Volumetric Data Processing and Interpretability

Facing the prevalent bottleneck of data scarcity in the medical imaging field, DETR, a model that typically requires large-scale data pretraining, faces severe challenges. Combining efficient learning strategies is key to overcoming this challenge. Utilizing self-supervised learning for pretraining on a large amount of unlabeled medical images to enable the model to learn domain-relevant general visual feature representations and then fine-tuning on downstream specific tasks are considered highly promising methods [131]. Simultaneously, combining DETR with semi-supervised learning frameworks, as demonstrated by Semi-DETR, which trains the model using a small amount of labeled data and a large amount of unlabeled data, is also an effective way to alleviate data scarcity.

Interpretability has always been a major obstacle to applying deep learning models in the medical field. DETR’s attention mechanism, particularly the cross-attention maps in the decoder, provides some possibility for understanding model decisions. By visualizing the image regions attended to by query vectors, it is possible, to some extent, to infer the image features on which the model bases its diagnosis [15]. This has positive significance for enhancing clinicians’ trust in AI-assisted diagnosis systems. However, interpretability based on attention maps is relatively superficial; the correlations they reveal are not necessarily equivalent to causality and may be somewhat misleading. Exploring more in-depth and reliable DETR interpretability methods is crucial for its safe application in clinical settings.

To meet clinical practical needs, extending DETR from 2D to 3D is an important research direction. Researchers have begun to explore applying the core ideas of DETR to 3D voxel data, which typically involves designing 3D convolutional backbones to extract voxel features and adjusting the Transformer’s attention mechanism to effectively handle 3D spatial dependencies, ultimately achieving end-to-end 3D lesion detection or anatomical structure localization [132]. Compared to traditional slice-by-slice 2D analysis or standard 3D CNNs (e.g., 3D U-Net, Mask R-CNN [133]), the advantage of a 3D-DETR lies in its global attention mechanism’s ability to capture long-range dependencies across slices, which is crucial for understanding complex 3D structures that span multiple slices. Furthermore, by adding a parallel mask prediction branch, the DETR framework can also be extended for 3D instance segmentation, similarly to the ideas in Mask R-CNN [133] and Mask DINO [90], providing new solutions for the precise delineation of anatomical structures or lesion contours.

### 4.3. Remote Sensing Image Analysis

Remote sensing image analysis plays an important role in numerous areas of the national economy and people’s livelihood, such as urban planning, resource monitoring, environmental assessment, and disaster response. Utilizing object detection technology to automatically extract objects of interest (e.g., aircraft, ships, vehicles, buildings, etc.) from high-resolution remote sensing images is one of the core tasks in this field. However, remote sensing images often exhibit characteristics significantly different from ordinary natural images, posing numerous challenges to the application of object detection algorithms:

1. Images are large in size and high in resolution, making direct processing computationally extremely expensive.
2. Object scale varies drastically, with objects spanning multiple sizes potentially coexisting in the same scene.
3. Small and dense objects are prevalent, such as dense building clusters, vehicles in parking lots, etc.
4. Object orientation is arbitrary, with many objects not aligned horizontally or vertically.
5. Backgrounds are complex and diverse, with rich types of ground objects, easily causing interference.

DETR and its variants offer new ideas for addressing these challenges.

#### 4.3.1. Handling Large-Size Images and Oriented Object Detection

For the problem of large image sizes in remote sensing, traditional methods often use sliding windows or patch-based processing, but this can easily cut objects at the patch edges and lose global context. To address this challenge, researchers have explored various solutions adapted for DETR. Some works mitigate the issue of object splitting to some extent using patch-based processing combined with overlapping region strategies [134]. Other more advanced methods leverage the linear complexity attention of Deformable DETR or combine it with the windowed attention mechanism of Swin the Transformer [135] to better balance global context information and computational overhead when processing large feature maps, showing potential superiority to simple patching.

Oriented Object Detection (OOD) is another core challenge in the remote sensing field. Traditional OOD methods are mostly based on CNNs, implementing it by introducing rotated anchor boxes or regressing angle parameters on horizontal proposals, such as the RoI Transformer. These methods often generate a large number of redundant proposals and rely heavily on Rotated NMS for post-processing, which is not only slow but also prone to suppressing correct results in dense scenes, such as ships packed in a harbor. DETR shows a paradigmatic advantage here: Its NMS-free nature is inherently suitable for handling dense objects. Researchers have successfully applied DETR to OOD tasks by extending its bounding box prediction head to additionally regress angle parameters and using Rotated IoU (RIoU) or its variants as the matching and regression loss [136]. For example, QEDetr [137] not only introduces angle encoding but also proposes Rotation-Aligned Deformable Attention (RADA), which allows attention sampling points to better adapt to the shape of rotated objects, thereby improving feature extraction accuracy. The comparative advantage over traditional methods is that DETR can directly output a refined, non-overlapping set of rotated boxes in an end-to-end manner, avoiding complex post-processing and potential suppression issues. The challenge is that, compared to mature CNN-based rotational detectors, DETR-based OOD models can be more complex to train, may converge more slowly, and can be more sensitive to hyperparameter settings.

#### 4.3.2. Detecting Small and Dense Objects and Task-Specific Optimization

Accurately detecting small and dense objects in remote sensing images is a core difficulty in the field. Although traditional CNN detectors use FPN to handle scale variation, their pre-defined anchors may not effectively cover the extreme range of object sizes in remote sensing images. More importantly, their reliance on NMS becomes a performance bottleneck when dealing with dense objects. DETR and its variants offer a different approach to these challenges. Introducing multi-scale features (e.g., via additional FPN layers [138] or using Deformable DETR) is a general strategy to enhance the feature representation of small objects. Additionally, researchers have explored more targeted methods. For instance, some works enhance performance by designing attention modules that focus more on high-resolution features [139] or by exploring dynamic query generation and allocation strategies tailored for remote sensing scenarios [140]. This allows queries to adaptively adjust based on the object distribution in the images, a more intelligent approach than the fixed anchor tiling of traditional detectors. For dense objects, DETR’s NMS-free characteristic provides a fundamental paradigmatic advantage. However, in practice, the one-to-one matching can still suffer from ambiguity and insufficient instance differentiation in extremely dense scenes, which requires improvements in matching strategies or enhanced interaction between queries. Ultimately, combining these general improvements with specific task requirements is key to achieving SOTA performance. For example, in specific applications such as aircraft detection in airports [137,141], ship detection in ports [105], and building extraction and change detection [140], researchers usually need to make targeted adjustments and optimizations to the model structure, training data (e.g., using spectral information), and loss functions, tailoring to the unique characteristics of the task.

### 4.4. Other Frontier Application Explorations

In addition to the aforementioned key areas, the flexibility and powerful representation capabilities of the DETR framework also demonstrate its potential in other diverse applications. How to apply it to new problem scenarios is also a direction actively explored by researchers.

#### 4.4.1. Pedestrian Detection

In pedestrian detection, especially in crowded scenes, traditional CNN-based methods are prone to suppressing correct detection boxes due to the high overlap of objects. DETR’s set prediction and one-to-one matching mechanism avoid NMS, providing a new approach for handling dense crowds. Furthermore, its self-attention mechanism among object queries also helps the model understand the relationships between individuals, reducing duplicate predictions. To further optimize detection performance in crowded scenes, some researchers, based on Deformable DETR, have proposed a progressive prediction method [142] to address the issues of duplicate predictions and inefficiency in crowded scenes. Other works have introduced an asymmetric relationship fusion mechanism to eliminate duplicate predictions and proposed decoupled cross-attention heads focusing on visible regions [143], effectively addressing duplicate predictions and occlusion issues in crowded scenes.

#### 4.4.2. Fine-Grained Visual Categorization

Fine-grained Visual Categorization (FGVC) tasks require models to distinguish between subcategories belonging to the same large category but with subtle differences (e.g., different car models). This typically requires the model to localize and focus on discriminative local regions in the image. DETR’s attention mechanism, particularly the cross-attention in the decoder, can learn to associate object queries with specific regions in the image. Therefore, some research explores using DETR and its variants to first localize objects or key regions and then extract features from these regions for subsequent fine-grained classification [144]. Alternatively, attention maps can be used as auxiliary information to guide classification decisions. This approach, combining localization and classification, has shown some improvement in FGVC performance, but how to more effectively utilize the features extracted by DETR for fine-grained discrimination still requires deeper exploration.

#### 4.4.3. Video Understanding

**Figure 19:**
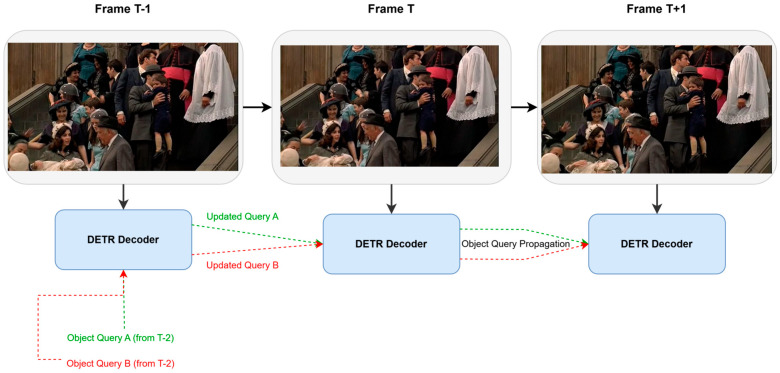
Conceptual pipeline for video object tracking via object query propagation.

DETR’s sequence processing capability also allows for its application in video understanding tasks. In video object detection and tracking tasks, object identity can be maintained across frames by associating object queries. For example, Trackformer [145] extends DETR to multi-object tracking by passing and updating queries in the temporal dimension to achieve tracking. The conceptual process of this mechanism, which maintains instance identity by propagating object queries across the temporal dimension, is illustrated in Figure 19. For each frame, the object queries from the previous frame serve as input to the current frame’s decoder, enabling the model to associate and continuously track objects over time. In Temporal Action Localization (TAL [146]) tasks, video frame sequences can be treated as inputs, using a Transformer encoder to capture spatiotemporal context information and employing a DETR-like query mechanism to identify and localize action instances occurring in video segments [147]. This is also a potential approach for video object tracking. Video instance segmentation can also be achieved in a similar manner; for example, Seqformer introduces a recursive structure to process temporal information. The key to applying DETR to video tasks lies in effectively modeling temporal dependencies and handling the higher computational complexity introduced by videos.

#### 4.4.4. Industrial Defect Detection

Industrial defects often exhibit characteristics such as irregular shapes, tiny sizes, low contrast, and diverse categories, and they frequently appear on surfaces with complex textures and reflective properties. These factors make it difficult for traditional methods based on template matching, fixed feature extraction, or simple threshold segmentation to achieve ideal results. DETR’s end-to-end learning paradigm and global context modeling capabilities offer new solutions for this. Its end-to-end training allows the model to directly learn defect features from data without complex feature engineering or predefined defect templates. The Transformer’s global attention mechanism also helps the model understand the relationship between defects and the surrounding background environment, thus better distinguishing between real defects and artifacts in complex textured backgrounds. Furthermore, DETR’s localization ability helps accurately mark the position and extent of defects, providing a reliable basis for subsequent quality control and repair. Some research has applied DETR and its variants to specific industrial defect detection tasks. For example, for defect detection on Printed Circuit Boards (PCBs), some works have used Deformable DETR to process PCB images, leveraging its multi-scale feature processing capability to detect defects of different sizes [148]. Researchers have also explored applying DETR to steel surface defect detection [149], textile defect detection [150], and other scenarios, utilizing DETR’s powerful feature learning and context understanding capabilities to identify various complex industrial defects. In these applications, it is usually necessary to fine-tune the model and perform data augmentation specifically for the particular defect types and imaging conditions.

Although the core architectural paradigm of DETR can effectively address challenges in different application domains, demonstrating its broad application potential as a flexible and powerful visual perception foundation framework, in many specific domains, the application of DETR is still in a continuous development stage, facing challenges in data (especially high-quality annotated data), efficiency, robustness, and interpretability, which also leaves ample space for future research.

To more clearly illustrate the overall picture of DETR’s applications in different specific visual perception domains and its technical adaptation, Table 5 summarizes the main challenges in each domain, corresponding DETR improvement strategies, and representative work.

## 5. Advanced Challenges and Future Research Directions

Building on the advancements detailed in Section 3, this section explores the deeper challenges that emerge as the field pushes toward more demanding applications. This progression naturally leads to revisiting topics like “small object detection” and “efficiency”, but from an advanced standpoint. The discussion, therefore, progresses from the foundational improvements to a deeper examination of these same topics, focusing on the remaining complexities and future research avenues for achieving further breakthroughs, particularly in extreme edge-case scenarios where current models still show limitations.

### 5.1. Toward Extreme Efficiency: A Roadmap for Edge Deployment

As discussed in Section 3.3, although models like RT-DETR have brought DETR into the realm of real-time detection, the concept of “real-time” varies significantly across different platforms. The core future challenge is achieving high-performance detection on edge devices with extreme power and cost constraints (e.g., ARM CPUs, mobile GPUs, NPUs). This requires a clear technical roadmap rather than simple compression of existing models. Future research should aim for specific quantitative targets: for example, achieving an inference speed of over 100 FPS on a typical mobile ARM processor, with a model size of less than 10 MB, while maintaining an accuracy of 40+ AP on benchmarks like COCO. Table 6 outlines this proposed roadmap, detailing the objectives, key research topics, and evaluation metrics for each phase.

Achieving these ambitious goals will require synergistic innovation along several technical routes. One promising path is Hardware-Aware Neural Architecture Search (NAS) [108], which uses actual hardware latency, rather than GFLOPs, as a direct optimization objective. A more challenging route involves extreme quantization and binarization exploration, investigating the impact of 4-bit or even 2-bit integer quantization on DETR models and exploring the feasibility of applying Binarized Neural Network (BNN) [85] concepts to the DETR framework. Ultimately, algorithm-hardware co-design will be crucial for achieving peak performance. This involves designing novel attention mechanisms that are inherently efficient for hardware, such as integer-only variants that avoid complex Softmax calculations, and co-designing them with specialized hardware accelerators, a direction explored by works like SpeedDETR [86]. Furthermore, lightweight designs for specific resource-constrained environments, such as DETR models optimized for thermal infrared object detection, also show great potential in this direction [151].

### 5.2. Overcoming the Final Hurdles in Small Object Detection

While the integration of multi-scale features, as reviewed in Section 3.1, provided a foundational solution by addressing the original DETR’s architectural deficiencies, fundamental challenges in feature representation persist. Specifically, for objects with extremely low resolutions (e.g., pixel area < 10 × 10) or poor signal-to-noise ratios, robust detection remains a significant concern. This is no longer just a matter of feature fusion but of fundamentally enhancing the model’s ability to perceive weak signals. Future research directions should include the following: exploring the integration of super-resolution techniques as a pre-processing module into the DETR pipeline to “magnify” small object features without significantly increasing computational cost; designing more sophisticated data augmentation strategies specifically for generating realistic small object samples at various scales; and researching novel attention mechanisms, such as variable-precision attention that can adaptively switch to higher computational precision in small object regions. Additionally, the deeper integration of denoising concepts with Feature Pyramid Networks (FPNs), as attempted by Denoising FPN [152], may also provide new ideas for filtering background noise and enhancing the signals of tiny objects.

### 5.3. Enhancing Generalization and Reliability in Open Environments

The training stabilization methods discussed in Section 3.2, such as denoising, have greatly improved model convergence and performance on benchmark datasets. However, real-world application environments are far more complex than benchmark datasets, and models need to be capable of working stably in various unknown, interfering, and even adversarial environments. Improving generalization and reliability is key for DETR to move from the laboratory to broader practical applications. The performance of current models in handling Out-Of-Distribution data, Long-Tail Recognition, adversarial attacks, and various data noises still needs improvement. Future research can explore the following: more effective self-supervised and semi-supervised learning paradigms to learn more robust visual representations from massive amounts of unlabeled data [131]; stronger Domain Adaptation [104] and Domain Generalization strategies to reduce performance degradation when deploying to new scenarios [153]; and DETR for Open World and Continual Learning, enabling it to recognize new categories and avoid catastrophic forgetting [99,100].

### 5.4. Deepening Theoretical Understanding, Interpretability, and Reliability

Empirical success requires the support of theoretical foundations and credible explanations. Although DETR has achieved empirical success, theoretical analyses of its internal working mechanisms by researchers are still insufficient. Future research should aim to establish a more solid theoretical foundation, for example, by attempting to build mathematical models to describe the information aggregation mechanisms of the Transformer’s self-attention at different visual scales or by analyzing the stability of bipartite matching from an optimization theory perspective. At the same time, enhancing model interpretability is key to improving user trust and facilitating model debugging. Although attention map visualization [15] provides some insight, it is often superficial and can be misleading [154]. Future research needs to more systematically apply advanced interpretability techniques to DETR, such as gradient-based methods (e.g., Grad-CAM [155]) or perturbation-based methods (e.g., LIME [156] and SHAP [157]) that are adapted for the Transformer architecture. Furthermore, improving model reliability is crucial. Works like E-DETR [102] have begun to explore quantifying prediction uncertainty using Evidential Deep Learning [103], while Cal-DETR [158] focuses on model calibration. Combining methods like Bayesian Deep Learning [159] to systematically evaluate and mitigate potential performance biases across different groups or conditions is an essential path to building more trustworthy and fairer DETR models.

### 5.5. Exploring Synergy with Multimodal and Other Frontier Technologies

Furthermore, expanding the application boundaries of DETR and exploring synergy with other technologies is full of opportunities. More tightly integrating the ideas of DETR with multimodal learning, such as fusing visual, linguistic, and point cloud information, holds promise for building more comprehensive perception systems [109,110,111]. Exploring the combination of DETR with generative models (GANs and Diffusion Models) may play a role in data augmentation, controllable image generation/editing, and even new visual reasoning tasks. Combining DETR with reinforcement learning for embodied AI tasks such as robotic active perception, grasping, and navigation is also a promising direction. Finally, designing more compact DETR structures for specific tasks, such as models for mask-wearing detection [160], also shows its great potential in vertical applications.

In essence, the cross-disciplinary explorations underscore a critical trend: DETR is evolving beyond a mere object detector into a versatile foundational framework for perception and reasoning. Its core components are the query mechanism and the attention-based feature fusion. It provides a flexible and powerful paradigm that can be adapted to a wide array of tasks. The synergy with other advanced AI technologies will undoubtedly unlock new capabilities and accelerate the development of more integrated and intelligent systems.

### 5.6. Strategic Overview of Synthesis

Having explored the specific advanced challenges in Section 5.1, Section 5.2, Section 5.3, Section 5.4 and Section 5.5, this subsection concludes Section 5 with a consolidated strategic overview. To synthesize these future directions and provide a structured outlook, Table 7 evaluates each direction based on its perceived priority, estimated technical difficulty, and potential data requirements.

The assessment in Table 7 provides a high-level strategic landscape. It suggests that the most immediate and high-impact challenges lie in enhancing efficiency and small object detection capabilities. These are not merely algorithmic problems but also resource challenges, potentially requiring new community efforts in dataset creation and hardware-specific benchmarking. In the medium term, improving generalization and reliability is paramount for transitioning these models from research prototypes to dependable, safety-critical products. Finally, while deeper theoretical understanding and synergy with other AI frontiers are ranked lower in immediate priority, they represent the long-term intellectual investments that will likely fuel the next paradigm shifts in the field. This strategic categorization helps frame the various research thrusts, clarifying the trade-offs between immediate practical needs and long-term foundational research.

## 6. Conclusions

The introduction of DETR marked a significant paradigm shift in the field of object detection. By innovatively combining the Transformer architecture with the idea of set prediction, it constructed a concise, elegant, and end-to-end detection framework that eliminates the need for hand-crafted post-processing like NMS, opening up a completely new path for subsequent research.

We systematically reviewed the development history of DETR since its inception from a “problem-driven” perspective. We first elucidated the fundamental theory and core architecture of DETR (Section 2) and then delved into the key technical breakthroughs and model evolution proposed by researchers to overcome the three major core challenges it initially faced: slow convergence, poor small object detection performance, and low computational efficiency (Section 3). These advancements include the introduction of efficient attention mechanisms, optimized object queries, and efficient architectural designs. These milestone works have greatly improved the performance, efficiency, and training feasibility of DETR.

Building upon this, the core ideas and framework of DETR have been successfully extended and applied to numerous specific visual domains (Section 4), including 3D perception and multimodal fusion in autonomous driving, lesion detection and segmentation in medical imaging, multi-scale and oriented object analysis in remote sensing images, and broader tasks like fine-grained visual categorization and video understanding, fully demonstrating its potential as a flexible perception paradigm.

However, despite significant progress, the technical ecosystem of DETR still faces critical challenges in practice, which can be quantitatively analyzed in terms of performance and resource requirements. First, the trade-off between performance and efficiency remains a central constraint. As detailed in Table 3, although advanced models like RT-DETR can achieve real-time inference speeds exceeding 100 FPS with a ResNet-50 backbone, this often comes at the cost of compromising on higher accuracy. When pursuing superior detection accuracy (e.g., upgrading the backbone to ResNet-101 to achieve 54.3 AP), the inference speed drops to approximately 74 FPS, while the computational load (GFLOPs) nearly doubles. This phenomenon indicates that achieving both top-tier accuracy and high frame rates on resource-constrained devices remains an open problem.

Furthermore, the limitation in small object detection persists. The original DETR achieved an AP for small objects (APs) of only 20.5 on the COCO dataset. Even for an SOTA model like RT-DETR-R50, the AP is 34.8. This figure stands in stark contrast to its performance on large objects (APl of 70.0), highlighting the ongoing challenge for the model to capture low-resolution features and fine spatial details. Finally, the resource threshold for model deployment cannot be overlooked. Even RT-DETR-R50, designed for real-time performance, requires over 40 M parameters and 136 GFLOPs. This level of computational and memory demand remains prohibitive for many edge computing devices with stringent constraints, such as drones and mobile robots.

These quantitative limitations, combined with the quantitative challenges in model robustness, interpretability, and reliability, define the key directions for future research (Section 5). We have reason to believe that with continued optimization toward extreme efficiency, enhancements of generalization and robustness in open environments, and a deeper theoretical understanding and technological synergy, DETR technology will continue to evolve and make greater contributions to advancing the capabilities of AI perception.

## Figures and Tables

**Table 1:** Overview of key DETR variants, core innovations, and addressed challenges.

Model	Year	Main Innovations	Core Problems Solved
DETR [22]	2020	Architecture: introduced transformer encoder–decoder for end-to-end object detection. Design: eliminated NMS and anchor boxes.	Simplified object detection pipeline by removing handcrafted components.
Deformable DETR [11]	2021	Attention Mechanism: introduced deformable attention to focus on key sampling points. Performance: improve efficiency and small object detection.	Addressed slow convergence and limited feature resolution of origin DETR.
Conditional DETR [38]	2021	Query Design: introduced conditional spatial queries for cross-attention. Training: speed up training convergence	Solved slow training convergence of DETR.
DAB-DETR [48]	2022	Query Design: used Dynamic Anchor Box as Queries. Training: improved convergence speed.	Solved slow training convergence and performance limitations of DETR.
DN-DETR [28]	2022	Training Method: introduced denoising training with noisy ground truth boxes. Matching: stabilized bipartite matching.	Accelerated training convergence of DETR-like models.
DINO [29]	2023	Query Design: combined dynamic anchor boxes and denoising training. Performance: achieved SOTA results.	Improved upon earlier variants’ slow convergence and performance issues.
RT-DETR [49]	2024	Architecture: used efficient hybrid encoder and IoU-aware query selection. Real-time: designed for high-speed inference.	Achieved high-accuracy real-time object detection, reducing computational costs.
DEIM [50]	2025	Matching Strategy: introduced dense O2O matching to increase positive samples. Loss Function: proposed matchability-aware loss (MAL).	Addressed sparse supervision and low-quality matched in DETR, improving convergence and accuracy.

**Table 3:** Key model performance comparison of DETR (COCO dataset).

Models	Backbone	Epochs	AP	AP50	AP75	APs	APm	APl	Params (M)	GFLOPs	FPS
DETR [22]	ResNet-50	500	42.0	62.4	44.2	20.5	45.8	61.1	41	86	28
DETR- DC5 [22]	ResNet-50	500	43.3	63.1	45.9	22.5	47.3	61.1	41	187	12
Deformable DETR [11]	ResNet-50	50	43.8	62.6	47.7	26.4	47.1	58.0	40	173	19
Sparse DETR [27]	ResNet-50	50	46.0	65.9	49.7	29.1	49.1	60.6	41	121	23.2
Sparse DETR [27]	Swin Transformer	50	49.3	69.5	53.3	32.0	52.7	64.9	41	144	17.2
Conditional DETR [38]	ResNet-50	108	43.0	64.0	45.7	22.7	46.7	61.5	44	90	17.8
Conditional DETR [38]	ResNet-101	108	44.5	65.6	47.5	23.6	48.4	63.6	63	156	-
DAB- DETR [48]	ResNet-50-DC5	50	45.7	66.2	49.0	26.1	49.4	63.1	44	216	17.0
DAB- DETR [48]	ResNet-101-DC5	50	46.6	67.0	50.2	28.1	50.5	64.1	63	296	-
DN- DETR [49]	ResNet-50	12	43.4	61.9	47.2	24.8	46.8	59.4	48	195	13
DN- DETR [49]	ResNet-50	50	49.5	67.6	53.8	31.3	52.6	65.4	47	195	13
CF-DETR [71]	ResNet-50 + TEF	36	47.8	66.5	52.4	31.2	50.6	62.8	41	173	16
CF-DETR [71]	ResNet-101 + TEF	36	49.0	68.1	53.4	31.4	52.2	64.3	60	253	14
RT-DETR [50]	ResNet-50	72	53.1	71.3	57.7	34.8	58.0	70.0	42	136	108
RT-DETR [50]	ResNet-101	72	54.3	72.7	58.6	36.0	58.8	72.1	76	259	74
YOLOv3 [25]	DarkNet	300	37.0	58.9	39.3	20.5	41.2	49.0	62.0	70.7	51
YOLOv4 [72]	CSPNet	300	43.5	65.7	47.3	26.7	46.7	53.3	~64	140	62
YOLOv5l [73]	CSPNet	300	49.0	67.3	53.3	29.0	53.6	64.7	46.5	109	~99
YOLOX-L [74]	CSPNet	300	50.0	68.5	54.5	29.8	54.5	64.4	54.2	155.6	~69
PP-YOLOE [75]	CSPNet	~300	51.4	68.9	55.6	31.4	55.3	66.1	52.2	110.1	78.1
YOLOv8l [76]	Advanced CSPNet	~300	52.9	69.8	57.5	35.3	58.3	69.8	43.7	165.2	110.4
YOLOV10l [7]	Enhanced CSPNet	100	53.2	70.1	58.1	35.8	58.5	69.4	24.4	120.3	137.4

**Table 4:** Key sensitive hyperparameters for milestone DETR variants.

Model Variant	Key Sensitive Hyperparameters	Description and Significance
DETR [22]	Loss Weight ($\lambda_{cls},\lambda_{L1},\lambda_{GIoU}$)	Description: These weights balance the classification loss, L1 box loss, and GIoU loss. Significance: Their configuration is critical as it directly impacts the balance between classification accuracy and localization precision. Incorrect balancing is a primary reason for slow convergence.
Deformable DETR [11]	Number of Sampling Points (K)	Description: The number of key sampling points attended to by each query in the deformable attention mechanism. Significance: A core parameter that trades off computational efficiency and performance. A smaller K leads to faster speed but may lose fine-grained details, while a larger K improves accuracy (especially for small objects) at a higher computational cost.
Conditional DETR [38]	Spatial Query Transformation	Description: Parameters of the FFN that generate the conditional spatial query from the 2D reference point. Significance: Controls the degree of spatial conditioning. This decoupling of content and spatial queries is key to accelerating convergence. Tuning this helps the model learn localization and recognition tasks more efficiently.
DAB-DETR [48]	BBox Update Step Size (or Learning rate)	Description: The step size for iteratively refining the 4D anchor box parameters (x,y,w,h) in each decoder layer. Significance: Directly controls the convergence of the box regression process. An appropriate step size ensures stable and progressive refinement of box predictions, which is the core mechanism of DAB-DETR.
DN-DETR [28]	Denoising Loss Weight ($\lambda_{dn}$)	Description: The weight of the auxiliary denoising task, which reconstructs ground-truth boxes from noised versions. Significance: This parameter controls the strength of the denoising supervision. A proper value is crucial for stabilizing the bipartite matching process and accelerating convergence, which is the core innovation of this model.
DINO [29]	Contrastive Denoising Noise Scale	Description: The magnitude of noise applied to create positive and negative samples for Contrastive Denoising Training (CDN). Significance: The noise scale defines the difficulty of the contrastive task. It must be tuned to compel the model to learn a precise boundary between true objects and near-negatives, directly improving localization accuracy and reducing duplicates.
RT-DETR [49]	Number of Initial Queries (K)	Description: The number of Top-K queries selected by the Uncertainty-Minimal Query Selection module to be fed into the decoder. Significance: This is a key parameter for balancing inference speed and accuracy. A smaller K significantly reduces the computational load in the decoder, enabling real-time performance, but may risk overlooking some objects in dense scenes.
DEIM [50]	Matchability-Aware Loss (MAL) Parameters	Description: Hyperparameters within the Matchability-Aware Loss function, which modulates the loss based on the quality of the match. Significance: These parameters directly influence how the model prioritizes high-quality matches during training. Fine-tuning them is essential for improving positive sample density and match quality, which addresses the core issue of sparse supervision in DETR.

**Table 5:** Summary of DETR applications in specific visual perception domains.

Application Domains	Key Domain Challenges	DETR Adaptations and Improvement	Representative Works/Models
Autonomous Driving	Real-time, Robustness, 3D Perception, Multi-modal Fusion	3D DETR, Fusion Strategies, Efficiency, Handling Occlusion/Density	DETR3D [117], BEV-Former [91], BEV-Fusion [118]
Medical Image Analysis	Small lesion, Data Scarcity, 3D Data, Interpretability	Small Object DETR, 3D DETR, Efficient Learning (Self/Semi-supervised), Interpretability/Reliability	LN-DETR [129], E-DETR [102]
Remote Sensing Image Analysis	Large Images, Dense/Small/Oriented Object, Complex Background	Large Image Handling, Small/Dense Object Queries/Features, OOB	QEDetr [137], DETR-ORD [140]
Pedestrian Detection	Crowded Scenes, Occlusion, Small Objects	Matching/Query Strategies for Crowds, Small Object Improvements	Recurrent DETR [67], AD-DETR [143]
Fine-grained Visual Categorization	Subtle Differences, Local Region Localization	Attention for Key Region Localization, Feature Extraction for Classification	Interpretable Transformer [144]
Video Understanding	Temporal Info, Object Identity, Action Localization	Processing Frame Sequences, Spatiotemporal Attention, Temporal Query, Crossframe Association	Trackformer [145], DITA [147]
Industrial Defect Detection	Irregular/Tiny Defects, Low Contrast, Complex Background	Feature/Matching for Defect, Global Context, End-to-End Detection	PCB Defect [148], Steel Defect [149], Textile Defect [150]

**Table 6:** A conceptual roadmap for achieving extreme efficiency.

Phase	Projected Milestone Dates	Potential Objectives and Research Topics	Suggested Evaluation Metrics (Targets)
Phase 1: Foundational Optimization	2025–2026	Advanced Quantization: further developing robust 8-bit/4-bit Post-Training Quantization (PTQ) and quantization-aware training (QAT) schemes for DETR. Efficient Architecture Search: utilizing hardware-aware NAS to optimize backbones and decoder layers for latency on mobile CPUs/GPUs.	Model Size: <20 MB; Latency (ARM CPU): <100 ms/frame. COCO AP: maintain > 45 AP.
Phase 2: Aggressive Compression	2026–2028	Extreme Quantization: exploring the feasibility of binary/ternary networks (BNN/TNN) for the most computationally intensive modules of DETR. Structural Pruning: designing algorithms for structured pruning of attention heads and FNN layers with minimal accuracy loss.	Model Size: <10 MB; Latencty (ARM CPU): <50 ms/frame. COCO AP: maintain > 40 AP.
Phase 3: Algorithm–Hardware Co-design	2028+	Integer-Only Attention: investigating novel, hardware-friendly attention mechanisms that may avoid softmax and floating-point operations. Co-Design with Accelerators: fostering collaboration with hardware engineers to design specialized NPUs or instruction sets for DETR-specific operations.	Model Size: <5 MB; Latencty (ARM CPU): <10 ms/frame. COCO AP: achieve > 40 AP with minimal power draw.

**Table 7:** A strategic assessment of future research directions.

Research Direction	Perceived Priority	Estimated Difficulty	Anticipated Data Requirements	Rationale
Extreme Efficiency	High	High	While algorithm development can start on standard benchmarks (e.g., COCO), extensive hardware-specific validation is necessary.	This direction is critical for unlocking widespread, real-world deployment on edge devices, a major current bottleneck.
Small Object Detection	High	Medium	Progress may be constrained by the limitations of existing datasets. New benchmarks with higher resolution and denser, smaller objects could be required.	This addresses a persistent performance gap in DETR-like models, limiting their applicability in domains like aerial imagery and medical analysis.
Generalization and Reliability	Medium	High	Research requires new evaluation protocols beyond AP (e.g., metrics for Out-of-Distribution robustness, calibration, fairness). Large-scale, diverse, and unlabeled data is beneficial.	This is crucial for building trust in safety-critical applications, though ensuring robust performance in open-world settings remains a formidable challenge.
Interpretability and Theory	Low	Very High	Foundational work can be performed on existing datasets, but a deeper understanding likely necessitates new analytical tools and theoretical frameworks.	While perhaps less urgent for immediate performance gains, this is important for long-term trust, debugging, and scientific advancement.
Synergy with Frontier Tech	Low	Very High	Progress likely depends on the availability of specialized, often multi-modal or simulation-based datasets (e.g., Vision Question–Answer, robotic interaction data).	Represents the long-term potential of DETR as a general perception module, but is highly exploratory and may require fundamental breakthroughs in multiple fields.

## Abbreviations

The following abbreviations are used in this manuscript:

DETR	DEtection TRansformer;
CV	Computer Vision;
AI	Artificial intelligence;
RPN	Region Proposal Network;
NMS	Non-Maximum Suppression;
NLP	Natural Language Processing;
CNN	Convolutional Neural Network;
ViT	Vision Transformer;
MSA	Multi-Head Self-Attention;
FFN	Feed-Forward Network;
LN	Layer Normalization;
MLP	Multi-Layer Perceptron;
IoU	Intersection over Union;
NLL	Negative Log-likelihood;
SOTA	State of the Art;
CDN	Contrastive Denoising Training;
RAQG	Ranking-Based Adaptive Query Generation;
SGL1	Soft Gradient L1 Loss;
AP	Average Precision;
GT	Ground Truth;
APs	Average Precision, small;
APl	Average Precision, large;
APm	Average Precision, medium;
BEV	Bird’s Eye View;
OVD	Open-Vocabulary Object Detection;
VLMs	Vision-Language Models;
FPN	Feature Pyramid Network;
OOD	Oriented Object Detection;
RADA	Rotation-Aligned Deformable Attention;
FGVC	Fine-Grained Visual Categorization;
TAL	Temporal Action Localization;
PCB	Printed Circuit Board;
CAVs	Concept Activation Vectors;
GANs	Generative Adversarial Networks.
